# Mismatch Repair–Proficient Colorectal Cancer Can Evade Immune Surveillance through an Intrinsic Suppressive Program

**DOI:** 10.1158/2159-8290.CD-26-0542

**Published:** 2026-05-13

**Authors:** Chiara Maria Cattaneo, Sharon Scardellato, Gianluca Mauri, Vittoria Matafora, Veera K. Ojala, Costanza Cannariato, Rosaria Chilà, Zulma Irene Magnani, Wouter Scheper, Luca Lazzari, Emile E. Voest, Maria Chiara Bonini, Angela Bachi, Salvatore Siena, Silvia Marsoni, Giovanni Germano, Alberto Bardelli

**Affiliations:** 1 IRCCS Humanitas Research Hospital, Milan, Italy.; 2 IFOM ETS–The AIRC Institute of Molecular Oncology, Milan, Italy.; 3 Department of Hematology, Oncology, and Molecular Medicine, Grande Ospedale Metropolitano Niguarda, Milan, Italy.; 4 Division of Immunology, Transplantation and Infectious Diseases, IRCCS San Raffaele Scientific Institute, Milan, Italy.; 5 Department of Molecular Oncology and Immunology, the Netherlands Cancer Institute, Amsterdam, the Netherlands.; 6 Oncode Institute, Utrecht, the Netherlands.; 7 Department of Oncology and Hemato-Oncology, University of Milan, Milan, Italy.; 8 Department of Medical Biotechnology and Translational Medicine, University of Milano, Milan, Italy.; 9 Department of Oncology, Molecular Biotechnology Center, University of Torino, Torino, Italy.

## Abstract

Although microsatellite-instable (MSI) colorectal cancers, reflecting mismatch repair (MMR) deficiency, often respond to immune checkpoint inhibitors, microsatellite-stable (MSS) tumors remain largely resistant. This disparity is typically attributed to differences in neoantigen load. However, whether antigen-independent mechanisms contribute to immune evasion in MSS colorectal cancer remains unclear. To address this, we engineered a model in which MSI and MSS colorectal cancer cells express identical levels of a defined antigen recognized by T-cell receptor–engineered T cells. Despite equivalent antigen presentation, MSS tumors exhibited impaired T-cell activation, reduced cytotoxicity, and resistance to killing. We linked this immune evasion to the MSS tumor secretome, which suppressed immune responses even in immunogenic MSI cells by impairing immune synapse formation. Surfaceome profiling by mass spectrometry identified glycosylation-dependent alterations that impair immune recognition. Our findings demonstrate that MSS colorectal cancer evades immune attack via intrinsic secretome-driven mechanisms, independent of antigenicity. Targeting glycosylation-linked suppressive pathways may restore T-cell responsiveness and improve immunotherapy efficacy in MSS colorectal cancer.

**Significance:**

This study reveals that MMR-proficient colorectal cancers resist T-cell attack not only due to low neoantigen load but also through glycosylation-dependent, secretome-mediated immunosuppression. These findings uncover a previously unrecognized immune evasion mechanism and identify actionable pathways to restore immunotherapy responsiveness in resistant colorectal tumors.

## Teaser

Microsatellite-stable colorectal cancer, even if presenting a strong, recognizable antigen, evades T cells through intrinsic, secretome-driven resistance, revealing antigen-independent mechanisms that limit immunotherapy efficacy.

## Introduction

Colorectal cancers can be classified based on the functional status of the DNA mismatch repair (MMR) system into two main subtypes: MMR-proficient (MMRp) and MMR-deficient (MMRd) tumors (1). In MMRd colorectal cancers, the inactivation of the MMR machinery is most commonly due to genetic or epigenetic alterations causing loss of function of the *MLH1*, *MSH2*, *MSH6*, and *PMS2* genes. This leads to genetic hypermutability and the accumulation of insertion–deletion mutations at microsatellite loci, a phenomenon known as microsatellite instability (MSI; 2). In colorectal cancer, MSI is therefore considered a hallmark feature of MMR deficiency. Conversely, MMRp tumors retain an intact MMR system and are characterized by microsatellite stability (MSS). MMRp/MSS tumors dominate the colorectal cancer landscape, representing approximately 85% of primary colorectal cancer cases. When considering the metastatic setting, their prevalence is even higher, accounting for nearly 95% of patients with colorectal cancer (3). For clarity and consistency with experimental readouts throughout this study, tumors are hereafter referred to according to their microsatellite status as MSI or MSS.

The advent of cancer immunotherapy, in particular the use of checkpoint inhibitors targeting programmed cell death protein 1 (PD-1) and cytotoxic T-lymphocyte–associated protein 4 (CTLA-4), has shown impressive clinical results in a subset of patients with solid tumors (4). Patients affected by metastatic MSI colorectal cancer often respond to therapies based on immune checkpoint blockade (ICB), whereas those with MSS tumors do not (5).

MSI tumors usually display high tumor mutational burden (TMB), a feature linked to rapid progression but also to favorable prognosis in colorectal cancer (6). Because of their high mutational burden, MSI tumors express a high number of neoantigens, novel protein sequences resulting from tumor-specific mutations that, when expressed on MHC class I (MHC-I) or II (MHC-II) molecules, may be recognized by patient T cells. According to the prevalent view, high TMB leading to elevated number of neoantigens increases tumor’s visibility to the T cell–based immune system (7). Levels of TMB are correlated with therapeutic response to ICB and, as a result, the US Food and Drug Administration (FDA) has granted tumor-agnostic approval for treatment with anti–PD-1 pembrolizumab for adult and pediatric patients with unresectable or metastatic solid tumors exhibiting a TMB higher than 10 mut/Mb (8, 9). Therapies based on checkpoint inhibitors are emerging as the standard-of-care treatment for MSI colorectal cancers (10–15).

With the exception of specific cases (e.g., *POLE*-mutated tumors), MSS tumors are instead characterized by a much lower number of mutations, and the prevalent view functionally links this feature to ineffective immunogenicity and lack of response to immune checkpoint inhibitors (ICI; 3). However, recent insights suggest a more nuanced picture. For instance, upon administering immunotherapy, MSS tumors show markers of immune activation, such as surge in CD8^+^ T cells or heightened IFNγ score, albeit with minimal (or absent) tumor regression (11). Moreover, with the goal of increasing the immunogenicity of MSS tumors, innovative clinical strategies have been implemented. For example, the alkylating agent temozolomide has been used to induce a “hypermutant” status in MSS patients, as a priming treatment to boost the number of tumor’s neoantigens, prior to anti–PD-1 treatment (16–18). Yet, despite an increase in the TMB, only a subset of patients benefited from ICB, whereas the majority of the patients remained refractory to checkpoint inhibition. These results suggest that factors beyond neoantigen load may be involved in therapeutic resistance of MSS tumors to cancer immunotherapies (19). It is conceivable that during their development, colorectal cancer could have acquired active mechanisms to evade immune surveillance.

Elucidating the mechanisms behind the poor response of MSS tumors is the key to develop new therapeutic strategies. However, the complexity of the tumor–immune system interaction, consisting of many different intrinsic and extrinsic effectors, makes it difficult to functionally pinpoint the underlying mechanism(s) of resistance (20). To tackle this issue, we developed a model system whereby both MSI and MSS tumor cells express equal levels of a given antigen which is recognized by engineered T cells. Using this model, we tested the hypothesis that the lack of T cell–mediated tumor recognition that characterizes MSS colorectal cancer might be due to an intrinsic resistance to T-cell attack and not only due to the low mutational burden.

## Results

To test the potential impact of intrinsic characteristics of MSS tumors on immune system recognition, we developed a tightly controlled experimental platform (Fig. 1A). To ensure unbiased assessment, it was necessary to keep the tumor recognition conditions identical across all samples. To achieve this, peripheral blood mononuclear cells (PBMC) were isolated from a healthy donor, followed by sorting and transduction of the CD8^+^ T cells fraction with a T-cell receptor (TCR) of known specificity. This preparation assures consistent and reproducible recognition across all experiments. In parallel, patient-derived tumor organoids (PDO) from both MSI and MSS colorectal cancers were established and screened for expression of a specific MHC-I molecule, HLA-A2. This step ensured that an identical antigen of choice could be uniformly presented to the previously engineered T cells, allowing for a controlled comparison between MSI and MSS tumor recognition.

**Figure 1.**
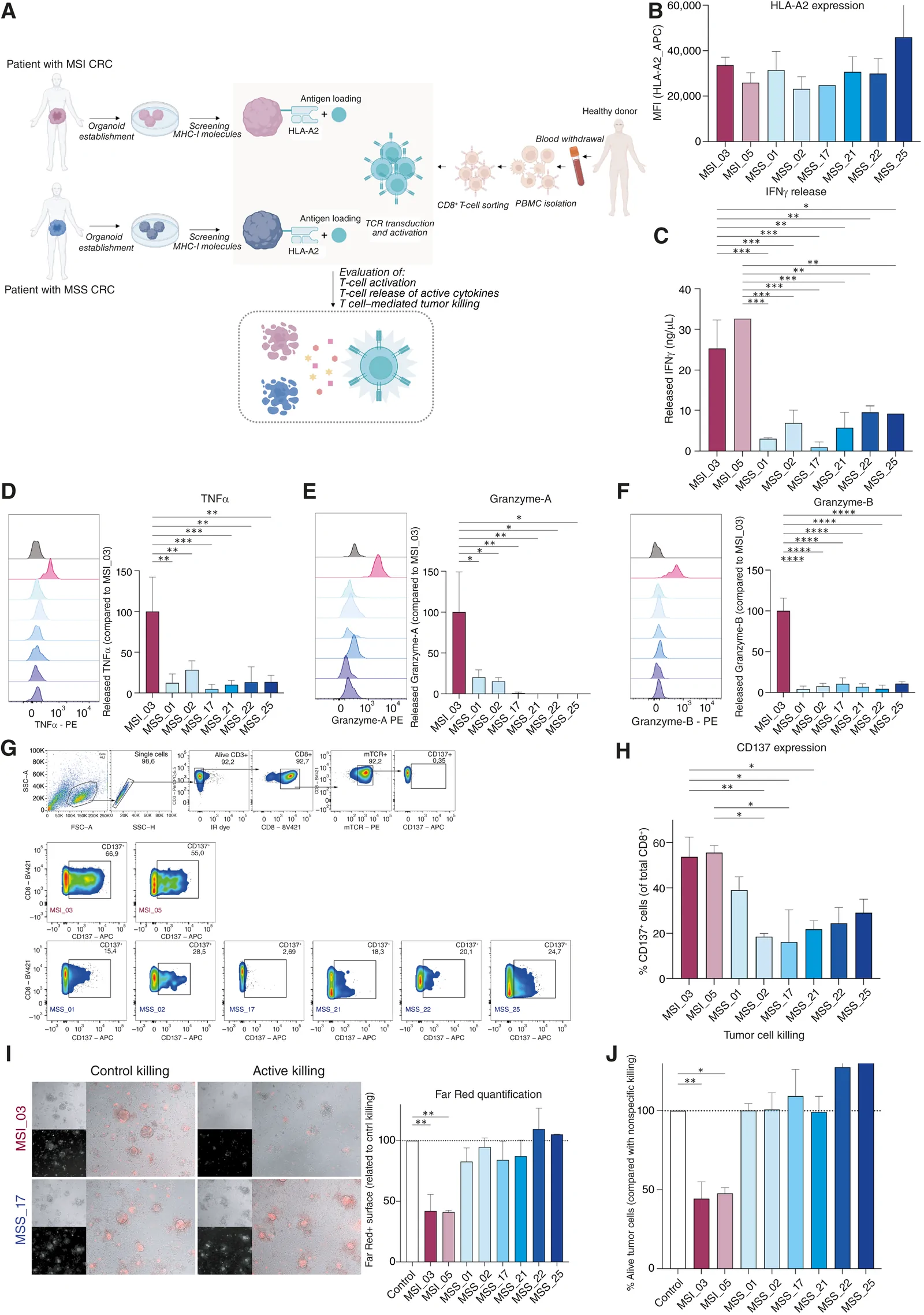
MSS colorectal tumors show intrinsic resistance to T-cell activity. **A,** Experimental workflow of the study. **B,** Cell-surface HLA-A2 expression in PDOs as determined by flow cytometry. Organoids were stimulated with 200 ng/mL IFNγ or left unstimulated. Bar graphs indicate the MFI. **C,** Quantification of organoid-induced IFNγ release in the supernatant. The background (spontaneous IFNγ production) was subtracted from the signal. **D,** Quantification of organoid-induced TNFα release (right). The background (spontaneous TNFα production) was subtracted from the signal. Left, representative histograms as determined by flow cytometry. The gray histogram represents unstained control. **E,** Quantification of organoid-induced granzyme-A release in the supernatant (right). The background (spontaneous granzyme-A production) was subtracted from the signal. Left, representative histograms as determined by flow cytometry. The gray histogram represents unstained control. **F,** Quantification of organoid-induced granzyme-B release in the supernatant (right). The background (spontaneous granzyme-B production) was subtracted from the signal. Left, representative histograms as determined by flow cytometry. The gray histogram represents unstained controls. **G,** Representative flow cytometry plots gated on live CD3^+^ CD8^+^ mTCR^+^ T cells, tested for reactivity against the indicated PDOs. Reactivity is measured as the percentage of CD137^+^ cells within the total mTCR^+^ T-cell population. **H,** Quantification of organoid-induced CD137 surface expression. The background (spontaneous CD137 expression) was subtracted from the signal. **I,** Left, representative pictures of the different PDOs 24 hours after coculture with T cells. Organoids were loaded (right) or not (left) with the MART-1_ELA_ antigen. Organoids were labeled with Far Red prior to coculture. On the right, quantification of tumor organoid killing, based on the Far Red signal. Data are normalized to the control condition (organoids cocultured with T cells without antigen), shown as the white bar. **J,** Quantification of PDO killing based on flow cytometry. At the end of the 24-hour coculture, tumor organoids were dissociated into single cells, and the number of live cells was quantified using counting beads. Data are normalized to the control condition (organoids cocultured with T cells without antigen), shown as the white bar. Error bars represent the SEM of at least two biological replicates. *, *P* < 0.05; **, *P* < 0.01; ***, *P* < 0.001; ****, *P* < 0.0001; ns, *P* > 0.05 (not shown on the graph); ordinary one-way ANOVA, multiple comparisons. (**A,** created in BioRender. Cattaneo, C. (2026) https://BioRender.com/zlfa7tr). MFI, median fluorescent intensity.

We successfully established and cultured human tumor organoids from surgical specimens obtained from a cohort of 32 patients with either MSI (*n* = 6) or MSS (*n* = 26) colorectal cancer tumors (Table 1). Immunohistochemistry (IHC) assessments confirmed the MMRd/MSI or MMRp/MSS profile of these organoids, based on the detection of MLH1, MSH2, MSH6, and PMS2 expression, as shown in Supplementary Fig. S1. Tumor organoids screening for MHC expression revealed variable levels of surface expression of MHC-I and MHC-II molecules (Supplementary Fig. S2A and S2B). The evaluation was conducted both with and without prestimulation using IFNγ. The presence of the primary HLA allele of interest, HLA-A2, was tested and detected in 12 different PDOs (Supplementary Fig. S2C). Furthermore, we checked programmed cell death ligand 1 (PD-L1) expression levels upon IFNγ stimulation (Supplementary Fig. S3) to determine whether the addition of ICIs in subsequent experiments would be required.

**Table 1.** Main clinicopathologic features of the cohort of patients with colorectal cancer.

Patient ID	Sex	Age	Primary tumor location	Stage	Samples used for PDO generation	Biopsy/resection	MMR status
MSS_01	F	52	Left colon	II	Primary tumor	Resection	MMRp
MSS_02	F	62	Left colon	IV	Liver metastasis	Resection	MMRp
MSS_03	M	66	Rectum	IV	Liver metastasis	Resection	MMRp
MSS_04	M	63	Left colon	III	Primary tumor	Resection	MMRp
MSS_05	M	68	Right colon	I	Primary tumor	Resection	MMRp
MSS_06	F	57	Rectum	III	Primary tumor	Resection	MMRp
MSS_07	F	77	Left colon	III	Primary tumor	Resection	MMRp
MSS_08	M	48	Left colon	III	Primary tumor	Resection	MMRp
MSS_09	M	72	Left colon	I	Primary tumor	Resection	MMRp
MSS_10	F	76	Left colon	I	Primary tumor	Resection	MMRp
MSS_11	M	47	Left colon	IV	Liver metastasis	Resection	MMRp
MSS_12	M	69	Right colon	III	Primary tumor	Resection	MMRp
MSS_13	F	50	Left colon	I	Primary tumor	Resection	MMRp
MSS_14	M	79	Left colon	I	Primary tumor	Resection	MMRp
MSS_15	M	49	Rectum	II	Primary tumor	Resection	MMRp
MSS_16	M	71	Left colon	I	Primary tumor	Resection	MMRp
MSS_17	M	70	Left colon	I	Primary tumor	Resection	MMRp
MSS_18	M	22	Left colon	III	Primary tumor	Resection	MMRp
MSS_19	F	84	Left colon	IV	Liver metastasis	Resection	MMRp
MSS_20	F	76	Rectum	III	Primary tumor	Resection	MMRp
MSS_21	M	81	Right colon	II	Primary tumor	Resection	MMRp
MSS_22	F	65	Left colon	II	Primary tumor	Resection	MMRp
MSS_23	M	62	Left colon	III	Primary tumor	Resection	MMRp
MSS_24	M	79	Left colon	II	Primary tumor	Resection	MMRp
MSS_25	M	73	Right colon	IV	Primary tumor	Resection	MMRp
MSS_26	F	71	Right colon	III	Primary tumor	Resection	MMRp
MSI_01	F	51	Right colon	IV	Lymph node metastasis	Biopsy	MMRd
MSI_02	F	59	Right colon	III	Primary tumor	Resection	MMRd
MSI_03	F	59	Left colon	III	Primary tumor	Resection	MMRd
MSI_04	F	85	Right colon	III	Primary tumor	Resection	MMRd
MSI_05	F	77	Left colon	IV	Peritoneal metastasis	Biopsy	MMRd
MSI_06	F	68	Right colon	IV	Primary tumor	Resection	MMRd

Abbreviations: F, female; M, male.

In parallel, we isolated CD8^+^ T cells from healthy donor PBMCs and retrotransduced them with the 1D3-TCR (MART1_25–35_-specific) antigen (Supplementary Fig. S4A; 21). After antibiotic selection and expansion, we tested antigen-specific activation capabilities by evaluating the number of CD137-expressing T cells in the presence (or not) of the target antigen MART-1_ELA_ (Supplementary Fig. S4B). Additionally, we confirmed the competence of T cells to react in the presence of the 12 HLA-A2^+^ PDOs (four MSI and eight MSS), when loaded with different concentrations of the Mart-1_ELA_ antigen, the MHC affinity–enhanced version of Mart-1_25–35_, which is specifically recognized by the transgenic TCR. T-cell activation was evaluated by measuring surface expression of CD137 and release of IFNγ (Supplementary Figs. S5 and S6, respectively).

To distinguish between the MSI and MSS tumors’ intrinsic capabilities of being recognized by the immune system, we selected organoids with comparable HLA-A2 expression levels and engineered them to present the same target antigen (MART-1_ELA_; Fig. 1B). Engineering of PDOs was obtained via peptide loading with a concentration of 0.02 µmol/L of MART-1_ELA_ antigen, thus preventing saturation across all samples. This decision was supported by the findings presented in Supplementary Fig. S5 and S6, where saturation was reached with 0.1 µmol/L of MART-1_ELA_ in several samples (MSS_16, MSS_17, MSS_21, MSS_22, MSS_24, and MSS_25 in Supplementary Fig. S5; MSI_03 and MSS_22 in Supplementary Fig. S6). Notably, when T cells were placed in the presence of MSI tumors, they secreted similar amounts of IFNγ. However, when the same T cells were exposed to MSS tumors, there was a consistent 60% to 85% reduction in IFNγ secretion (Fig. 1C). Similar secretion patterns were observed for other effector molecules, such as tumor necrosis factor α (TNFα; Fig. 1D; Supplementary Fig. S7A), granzyme-A (Fig. 1E; Supplementary Fig. S7B), and granzyme-B (Fig. 1F; Supplementary Fig. S7C). T-cell reactivity assessed by CD137 expression led to analogous results. In the presence of MSI tumors, TCR-transduced T cells showed comparable levels of CD137 expression. However, in the majority of the MSS PDOs, exposure to T cells led to a significantly diminished reactivity level (Fig. 1G and H). These observations held true also when the wild-type, non–MHC affinity–enhanced, variant MART-1_EEA_ was used (Supplementary Fig. S8A and S8B).

Next, a 24-hour killing coculture assay (22, 23) was used to assess how effectively the different PDOs were eliminated by T cells. Both Far Red signaling and flow cytometry–based quantifications were used to assess the amount of surviving tumor cells. As shown in Fig. 1I and J, MSI PDOs showed a notable reduction of the count of live cells (∼50%) compared with controls, whereas MSS PDOs remained largely unaffected, with no killing detected (Fig. 1I and J).

We reasoned that these results pointed to the existence of mechanisms which extend beyond mere mutational (neo)antigenic burdens, limiting the response of MSS colorectal cancer to ICB.

To explore the immunomodulatory milieu during antigen recognition, we profiled the supernatants from MSI and MSS tumor organoids, each loaded with the MART-1_ELA_ peptide and cocultured with autologous T cells, using a 96-plex immuno-oncology panel (Fig. 2A; Supplementary Fig. S9; 24). Comparison of MSI versus MSS cocultures revealed multiple statistically significant differences in protein abundance. In particular, hallmark effector cytokines of T-cell activation, including IFNγ and TNFα, were markedly elevated in the presence of MSI organoids, and a distinct array of chemokines and inflammatory mediators involved in immune cell recruitment and intercellular communication were differentially expressed—findings that agree with our earlier functional assays. Strikingly, further inspection of the significant hits revealed that several mediators were not solely T cell–derived but were also secreted by the tumor organoids themselves (Fig. 2A).

**Figure 2.**
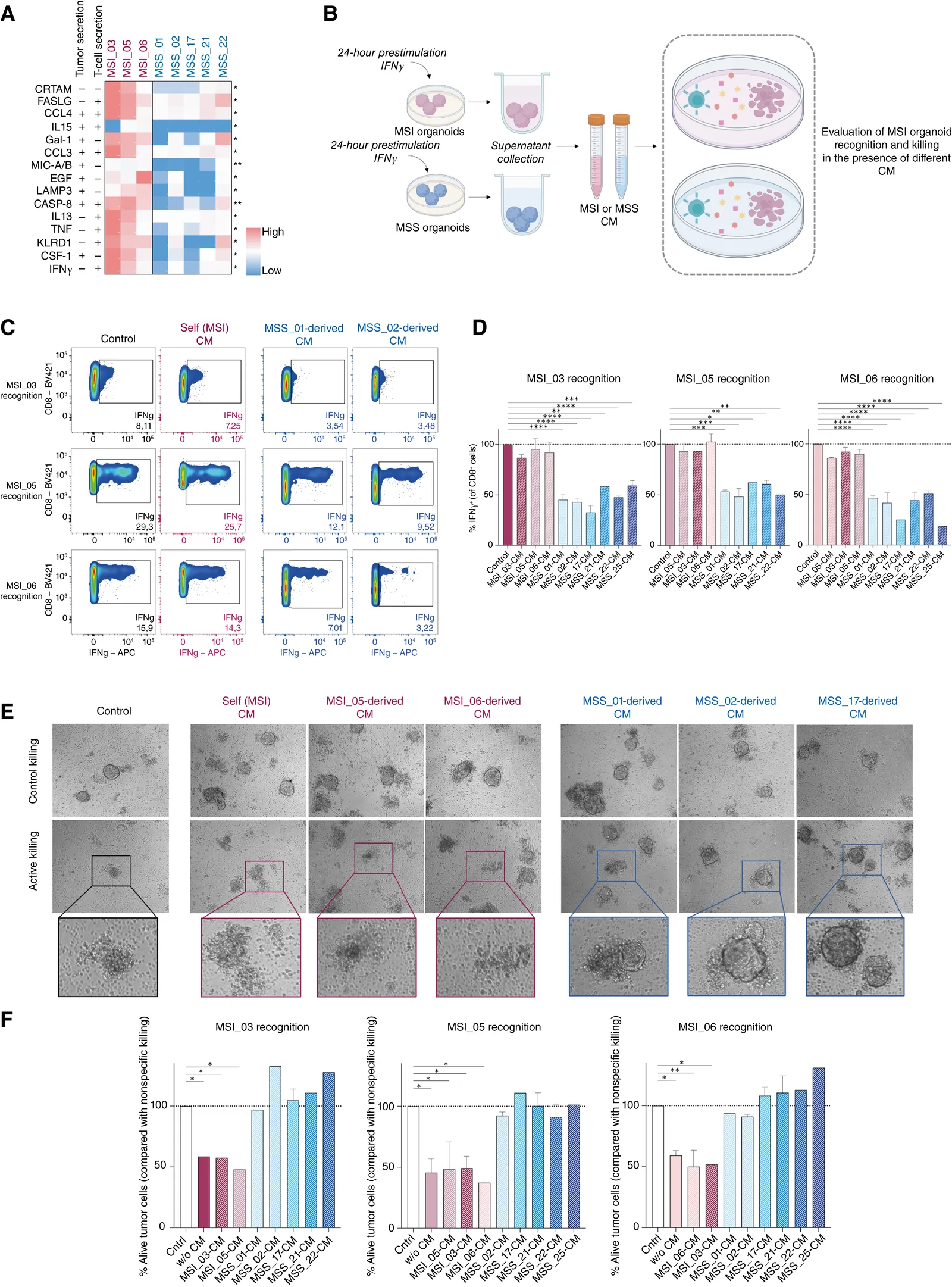
The tumor secretome is a key component of MSS intrinsic resistance to cancer immunotherapy. **A,** Heatmap showing relative expression levels of a panel of immunomodulatory molecules in the supernatant of MSS and MSI PDOs. Colors represent fold changes in immunomodulatory molecule levels (red indicates upregulation, whereas blue indicates downregulation). Columns represent individual patients. Each data point originates from at least three technical or biological replicates. Molecules and experimental conditions that have statistically significant abundance between MSS and MSI samples are marked. *, *P* < 0.05; **, *P* < 0.01; ns, *P* > 0.05 (not marked in the figure); Student *t* test. For each molecule, we marked whether they are usually secreted by tumor cells, immune cells, or both. **B,** Workflow of the experiment. **C,** Representative flow cytometry plots gated on CD8^+^ mTCR^+^ T cells, tested for reactivity against MSI tumor organoids in the presence of different CM. **D,** Quantification of IFNγ intracellular expression in T cells with MSI organoid in the presence of the indicated CM. The background (spontaneous IFNγ expression) was subtracted from the signal. The control condition (no CM) was used as reference for normalization. **E,** Representative pictures of MSI_03 PDOs 24 hours after coculture with T cells. Organoids were loaded (bottom) or not (top) with the MART-1_ELA_ antigen. The coculture was performed in the absence of CM (left, indicated in black), in the presence of different MSI PDO-derived CM (middle, indicated in purple), or in the presence of different MSS PDO-derived CM (right, indicated in blue). **F,** Quantification of MSI PDO killing in the presence of the indicated CM, based on flow cytometry. At the end of the 24-hour coculture, tumor organoids were dissociated into single cells, and the number of live cells was quantified using counting beads. Data are normalized to the control condition (organoids cocultured with T cells without antigen), shown as the white bar. Error bars represent the SEM of at least two biological replicates. *, *P* < 0.05; **, *P* < 0.01; ***, *P* < 0.001; ****, *P* < 0.0001; ns, *P* > 0.05 (not shown on the graph); ordinary one-way ANOVA, multiple comparisons. (**B,** created in BioRender. Cattaneo, C. (2026). https://BioRender.com/33ffulm).

This dual origin suggests the existence of tumor-intrinsic circuits that may contribute to the immune modulatory phenotype we observe. We therefore hypothesized that tumor-secreted factors play a direct role in shaping the secretome and may constitute an alternative mechanism of immunoresistance in our system.

To investigate whether factors secreted by colorectal cancer cells (the secretome) played a role in restricting their recognition by T cells, we utilized conditioned media (CM) from MSI and MSS PDOs, collected 24 hours after IFNγ stimulation (Fig. 2B). We set up a T-cell recognition assay which involved measuring intracellular IFNγ expression levels upon coculture with MSI tumor cells in the presence of PDO-derived CM. MSI tumor cell recognition was not affected by CM derived from MSI tumor organoids. Strikingly, when MSS-derived CM was used, T-cell recognition of MSI tumor cells was markedly impaired (>50% reduction) in all cases (Fig. 2C and D). The killing efficacy of T cells was similarly impaired in the same experimental conditions (Fig. 2E and F).

To further delve into the functional mechanisms constraining T-cell functionality, we evaluated the impact of PDO-derived CM on T cells by examining three key parameters: viability, proliferation, and target recognition capability. T-cell viability was determined using an ATP release assay. Following 24 hours of culture in the presence of different CM, no significant alterations in T cells’ viability were observed (Supplementary Fig. S10A). Next, T-cell proliferation, both unstimulated and stimulated by the MART-1ELA antigen, was measured by flow cytometry. The presence of CM from MSS tumor organoids did not significantly affect T-cell proliferation (Supplementary Fig. S10B). Finally, to assess T-cell target recognition, we utilized professional antigen-presenting cells (APC) loaded with the MART-1ELA peptide. T cells were cocultured for 5 hours with the APCs in the presence of different CM, and intracellular IFNγ expression was measured. Consistently with previous data, no significant differences were observed across the different CM used (Supplementary Fig. S10C).

These results show that the CM derived from MSI and MSS cocultures had no detectable impact on T-cell viability, proliferation, or activation, indicating the absence of a direct suppressive effect on T-cell functionality. Notably, professional APCs loaded with the same MART-1_ELA_ peptide were efficiently recognized by T cells even in the presence of tumor-derived CM. These findings suggest that the impaired recognition observed in tumor organoid–T cell cocultures is not due to a general inhibition of T-cell responsiveness but rather reflects a mechanism that specifically interferes with T cell–tumor cell interactions.

Given that the observed impairment was specific to tumor–T cell interactions, we next sought to functionally assess the quality of this interaction under different conditions. Specifically, we focused on two parameters: the degree of T-cell engagement and the strength of the resulting interactions, reflecting the stability and avidity of T cell–tumor contacts. These metrics allowed us to quantitatively evaluate how CM influences the formation and robustness of the tumor–T cell binding.

To quantify T-cell engagement with tumor cells, we performed high-content microscopy on cocultures of labeled T cells and tumor organoids. The proportion of T cells in direct contact with tumor cells was calculated and used as a measure of engagement efficiency across conditions (Fig. 3A). First, to assess whether tumor-intrinsic properties influence the ability of T cells to establish contact, independently of antigen presentation, we used this assay to compare T-cell engagement across different MSI and MSS tumor organoids, all presenting the MART-1_ELA_ antigen (Fig. 3B). Next, to isolate the impact of MSS tumor-derived secretome on the physical interaction between T cells and tumor cells, we focused on two different MSI tumors singularly and evaluated engagement in the presence of different CM (Fig. 3C). These experiments revealed that T-cell engagement varies significantly between MSI and MSS tumor organoids, despite presentation of the same cognate antigen. For MSI tumors, we observe that approximately 60% of T cells established contact with tumor cells, whereas with MSS tumors engagement dropped to as low as 20% (Fig. 3B). A similar reduction in engagement was seen when MSI tumor organoids were placed with T cells in the presence of MSS-derived CM, resulting in a decrease of approximately 50% in the proportion of engaged T cells (Fig. 3C).

**Figure 3.**
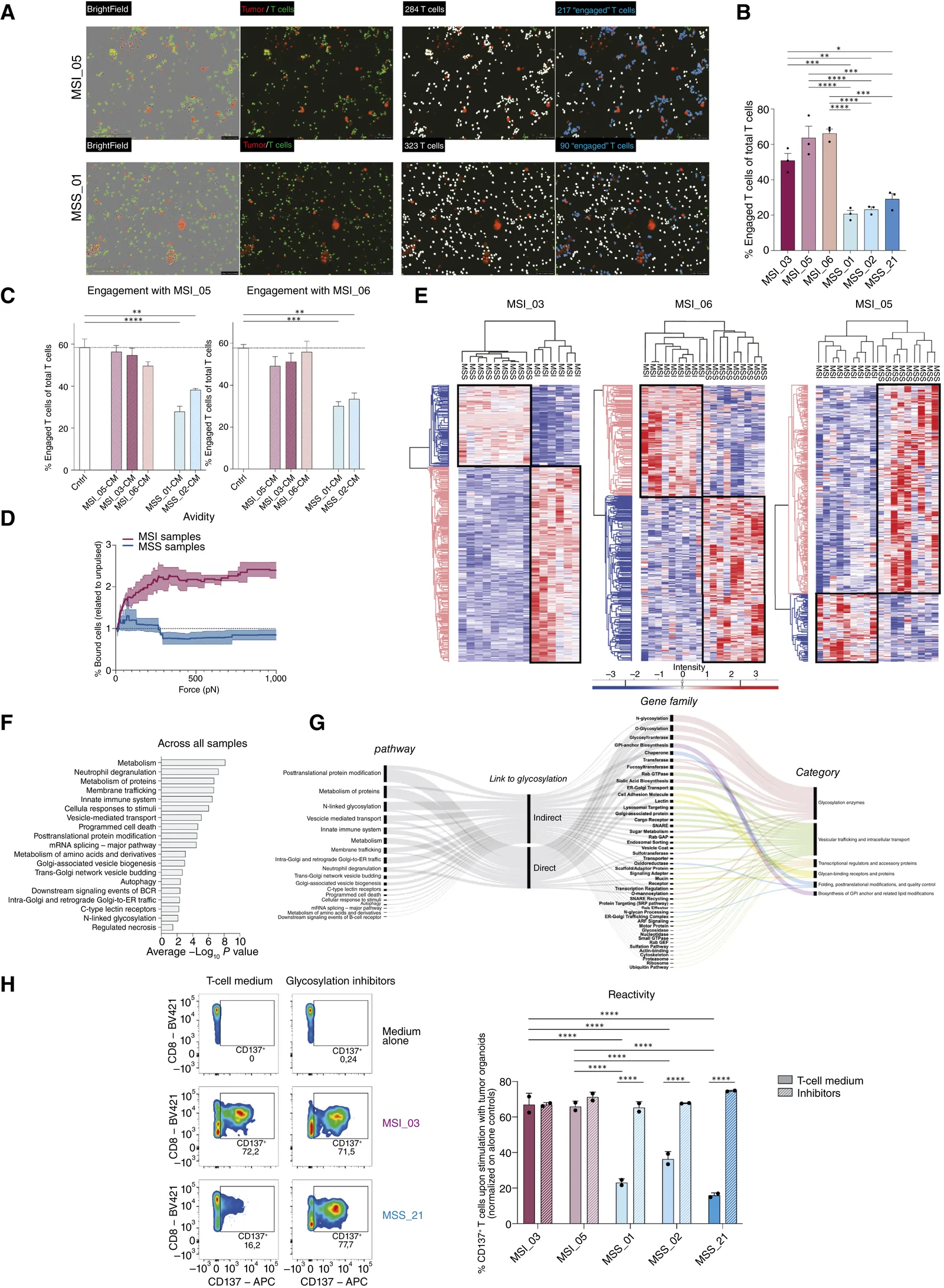
Functional analysis of the tumor secretome. **A,** Representative high-content microscopy images of MART-1_ELA_–loaded tumor organoids (red) cocultured with fluorescently labeled T cells (green). Total T cells are segmented and highlighted with a white mask, whereas T cells engaged in direct contact with tumor organoids are marked in blue. Engagement was quantified as the proportion of T cells physically interacting with tumor cells. The total number of T cells and the number of engaged T cells are indicated in each image. **B,** Quantification of engaged T cells across different PDOs, all loaded with MART-1_ELA_ antigen; MSI samples are indicated in pink, and MSS in blue. Error bars represent the SEM of at least two biological replicates. *, *P* < 0.05; **, *P* < 0.01; ***, *P* < 0.001; ****, *P* < 0.0001; ns, *P* > 0.05 (not shown on the graph); ordinary one-way ANOVA, multiple comparisons. **C,** Quantification of T-cell engagement with MART-1_ELA_- loaded MSI_03 tumor organoids (left) or MSI_05 tumor organoids (left) cultured in the presence of different CM. The control condition (no CM, showed in white) was used as reference. *, *P* < 0.05; **, *P* < 0.01; ***, *P* < 0.001; ****, *P* < 0.0001; ns, *P* > 0.05 (not shown on the graph); ordinary one-way ANOVA, multiple comparisons. **D,** Analysis of T-cell binding strength to PDOs from MSI (pink) and MSS (blue) tumors. Two distinct tumor organoids lines were used for each group. Data from each experiment were normalized to their respective unpulsed controls (organoids without MART-1_ELA_ peptide loading). The mean ± SEM of biological replicates are shown. Binding strength is represented as the percentage of T cells remaining attached to tumor cells under increasing applied acoustic force. **E,** Label-free membrane proteome quantification of MSI tumor organoids treated with MSS or MSI CM for 24 hours. Hierarchical clustering analysis (heatmap) using unsupervised Euclidean distance of differentially expressed proteins between MSI samples treated with MSS or MSI CM (Student *t* test, *P* value <0.05. *N* = 3 biological replicates). Each row in the figure represents a protein, and each column represents a sample. Red, high expression; blue, low expression. **F,** Annotations of proteins significantly upregulated across the different MSI tumor organoids treated with MSS CM. Reactome pathway enrichment analysis performed by the web tool Enrichr. Pathways categories and their relative average of enrichment scores (average −log_10_*P* value) were plotted as histogram, and pathways directly or indirectly linked to glycosylation are shown in black and gray, respectively. **G,** Alluvial plot illustrating the relationship between significantly enriched pathways and glycosylation-related genes. Significantly enriched pathways identified via proteomic analysis and Enrichr are shown on the left. Each pathway is connected to glycosylation-related genes that were significantly altered and fall within that pathway. The flow passes through two intermediate layers: the nature of the link with glycosylation (direct vs. indirect) and the glycosylation gene functions to which the altered genes belong. Flows then converge on broader macrocategories representing the functional classification of these genes. The thickness of the streams corresponds to the relative number of genes involved, with thicker streams indicating direct links to glycosylation. ER, endoplasmic reticulum. **H,** Left, representative flow cytometry plots gated on live CD3^+^CD8^+^ mTCR^+^ T cells cocultured with the indicated PDOs in the presence or absence of glycosylation inhibitors. T-cell reactivity is measured as the percentage of CD137^+^ cells within the total mTCR^+^ T-cell population. Right, quantification of organoid-induced CD137 surface expression in T cells cultured with the indicated PDOs in the absence (solid color) or presence (striped bars) of glycosylation inhibitors. Background signal (spontaneous CD137 expression) was subtracted from the measured values. Error bars represent the SEM from at least two biological replicates. ****, *P* < 0.0001; ns, *P* > 0.05 (not shown). Statistical significance was determined using ordinary one-way ANOVA with multiple comparisons.

To complement our engagement analysis, we next assessed the strength of T cell–tumor interactions using a single-cell force-based assay (25). This approach revealed marked differences in the stability of T cell–tumor contacts between MSI and MSS tumors, mirroring what we assessed with the engagement data (Fig. 3D). Notably, when the binding strength to MSI tumors was measured in the presence of MSS tumor-derived CM, we noticed a significant reduction (Supplementary Fig. S10D).

These findings suggest that MSS tumor-derived soluble mediators can modulate the efficiency of T cell–tumor interactions, impairing the quality of the immune synapse.

To understand the mechanisms behind our observation, we first analyzed the surface expression of four key molecules critical to our system. We exposed four different MSI tumor organoids to CM collected from both MSI and MSS tumor organoids. Following this exposure, we assessed the surface expression levels of MHC-I, HLA-A2, MHC-II, and PD-L1. No significant differences were observed in the expression of these molecules across the different CM treatments (Supplementary Fig. S10E).

We next sought an unbiased view of how tumor-derived secretomes remodel the tumor-cell surface. To this end, we performed mass spectrometry (MS)–based membrane proteome on MSI tumor organoids that had been preactivated with IFNγ and then cultured for 24 hours in the presence of CM from either MSI or MSS tumors. Unsupervised clustering of the resulting membrane–protein intensities revealed a striking separation between the two treatment groups (Fig. 3E), demonstrating that tumor-secreted factors profoundly reprogram surface-protein distribution (all detected proteins and statistically significant differences are reported in Supplementary Tables S1–S6).

Pathway analysis of differentially abundant proteins revealed that exposure to MSS CM leads to substantial molecular reprogramming (Fig. 3F; Supplementary Fig. S11A). Affected pathways were primarily associated with immune regulation, metabolic activity, and posttranslational modifications, suggesting that the MSS-associated secretome exerts a broad modulatory effect on mediators of immune cell function and intercellular communication. Among the altered pathways, we specifically investigated those that could reflect tumor-intrinsic mechanisms actively contributing to immune evasion within a short timeframe (5–24-hour-assay). Given the evolutionary conservation and the well-established role of glycosylation in shaping immune escape, remodeling the tumor microenvironment, and modulating T cell–mediated recognition and immunotherapy response (26–31), we focused on this process. We therefore analyzed the subset of genes that were both significantly altered and annotated within the enriched pathways, with particular attention to those functionally linked to glycosylation. This analysis is summarized in the alluvial plot (Fig. 3G), which illustrates the connection between enriched pathways and glycosylation-related genes, their respective gene functions, and broader functional macrocategories. Strikingly, a substantial proportion of the altered genes exhibited direct or indirect associations with glycosylation, underscoring the prominent role of this modification in mediating the proteomic effects of MSS-derived secreted factors. The intersection between significantly regulated membrane proteins and glycosylation-related pathways across individual PDOs is shown in Supplementary Fig. S11B.

To functionally validate the impact of glycosylation on immune recognition, we repeated the MSI T-cell recognition assay in the presence of CM from both MSI and MSS sources (Fig. 2B–D). In this setting, we cotreated organoids with the O-glycosylation inhibitor benzyl-α-GalNAc and the glycosyltransferase inhibitor lithocholic-O-aspartate (Supplementary Fig. S12). Remarkably, inhibition of glycan elongation fully restored T-cell recognition in all MSI samples, reaching levels comparable with untreated controls (Supplementary Fig. S12). These results demonstrate that tumor-derived soluble factors can induce glycosylation-dependent changes that directly interfere with T cell–tumor interface, potentially promoting immune escape.

To further determine whether glycosylation directly modulates tumor cell susceptibility to T-cell recognition, we performed additional coculture experiments in the absence of CM using antigen-loaded PDOs. Consistent with our previous observations, MSS organoids induced significantly lower T-cell activation compared with MSI organoids. Importantly, pharmacologic inhibition of glycosylation had minimal impact on MSI samples but restored T-cell activation in MSS organoids to levels comparable with MSI controls (Fig. 3H), confirming a direct role of glycosylation in limiting T-cell responsiveness in MSS tumors.

Cumulatively, these findings indicate that MSS colorectal cancers are capable of actively disrupting T-cell recognition and activation, revealing a novel tumor cell–intrinsic mechanism of immune escape. Our data identify the tumor secretome as a key regulator of immune recognition dynamics, specifically impairing T-cell engagement and the formation of a stable tumor–immune synapse. Surface proteomic profiling further demonstrated that tumor-derived factors extensively remodel the tumor membrane landscape, with glycosylation emerging as a central, functionally relevant modification. Together, these results highlight a previously underappreciated pathway of immune evasion mediated by secretome-driven modulation of the tumor-cell surface.

## Discussion

Patients diagnosed with MSI colorectal cancer often benefit from therapies based on ICB, whereas those with MSS tumors generally do not (5, 10, 11, 13). The prevalent view is that MSI tumors are recognized by the immune system owing to their elevated mutational/neoantigen repertoire. However, emerging evidence suggests that factors beyond neoantigen count play a pivotal role in modulating the therapeutic response. In the current study, we aimed to dissect the biological mechanisms responsible for the different immune response in MSS compared with MSI tumors.

To this end, we first developed an experimental platform wherein both MSS and MSI tumors presented the same antigen (MART-1_ELA_) at an identical concentration via the same presenting molecule (HLA-A2) to a homogeneous T-cell population engineered to express 1D3-TCR. This design ensures that the main variables in the tumor–host interaction are the intrinsic features of the cancer cells. MSS tumors consistently displayed a reduced recognition by the immune system. Impaired immune activation was accompanied by a common biological profile as defined by distinct effector molecules and activation markers such as IFNγ, TNFα, granzyme-A, granzyme-B, and CD137. Functional assessment of T-cell cytotoxic capacity in the presence of MSS and MSI cells revealed that although MSI tumors were efficiently killed by the engineered T cells, MSS tumors remained unaffected.

The experimental platform we implemented in this study can be broadly used for understanding how distinct cancer subtypes modulate T-cell reactivity. Moreover, these results show that MSS tumors are intrinsically recalcitrant to T cell–mediated recognition and killing. How tumor-intrinsic factors modulate tumor–host interaction, immune recognition, and the ensuing killing have remained largely unrecognized. Our findings have a strong clinical relevance: even if the TMB of MSS tumors was successfully augmented by experimental strategies, for example, with mutagenic/alkylating agents, the native resistance mechanisms of MSS tumors could still hamper an effective immune-mediated control, as shown in our current work. The same would apply if a strongly immunogenic neoantigen is successfully introduced in MSS colorectal cancer. In this regard, genetic or pharmacologic approaches designed to disable the MMR pathway rather than simply increasing the TMB could be advantageous, as we and others have shown (16–18, 32–34).

After establishing that tumor-intrinsic factors are instrumental for resistance and response to immune surveillance, we set out to characterize the biological basis for this phenotype.

We focused on the tumor secretome as a potential contributor to the observed differences. Proteomic profiling revealed distinct differences in the soluble factors released by MSI and MSS tumors during antigen-specific T-cell engagement. Notably, MSS tumors shaped a secretory milieu that seemed less supportive of immune activation, characterized by reduced lymphocyte recruitment and weakened proapoptotic signaling, suggesting a shift toward an immunosuppressive environment. To functionally test whether tumor-derived soluble factors directly affect T-cell activity, we performed secretome functional experiments. The results were striking: CM derived from MSS tumor organoids significantly reduced T-cell recognition of MSI tumor cells. Furthermore, exposure of engineered T cells to MSS CM was sufficient to impair their cytotoxic ability against MSI targets. These findings support the notion that MSS tumors engage tumor-intrinsic secretory programs capable of interfering with effective immune recognition.

Importantly, the effects mediated by MSS CM were not due to a generalized impairment of T-cell function. T cells retained their viability, proliferative capacity, and remained fully responsive when stimulated by professional APCs, even in the presence of tumor-derived soluble factors. This indicates that the observed phenotype is not the result of broad immunosuppression but rather reflects a mechanism that specifically interferes with the physical interaction between T cells and tumor cells.

By analyzing the dynamics of T cell–tumor engagement, we observed a reduced frequency and strength of T-cell contact in the presence of MSS tumors or MSS-derived CM, suggesting that the impaired recognition involves active modulation of the tumor–immune interface.

To further confirm our findings, we used an unbiased surface proteomic approach, which uncovered a widespread remodeling of the tumor membrane in response to MSS-derived factors. Among the pathways altered by MSS CM, we primarily observed changes in metabolism, immune-related molecules, antigen presentation, and cell–cell communication. Glycosylation, although not explicitly highlighted as a top enriched pathway in our analysis, emerged from the profile of proteins involved as a likely modification affecting key molecules at the immune synapse. Our data, together with previous reports emphasizing the importance of metabolic rewiring, especially glycosylation, in immune escape, support the hypothesis that this posttranslational modification reshapes the tumor-cell surface (30, 31, 35, 36). Indeed, glycosylation is an evolutionarily conserved mechanism through which tumor cells remodel their surface to evade immune detection, with aberrant glycan patterns shown to alter receptor–ligand interactions, modulate antigen presentation, and disrupt immune synapse formation across multiple cancer types (26–31). Our findings build on this established framework and uncover a previously unrecognized layer of complexity: in MSS colorectal cancer, tumor-secreted factors are sufficient to induce glycosylation-dependent surface remodeling on neighboring tumor cells, extending immune evasion beyond cell-autonomous mechanisms.

Given the known role of glycosylation in immune modulation, we tested its functional relevance in our system. Strikingly, pharmacologic inhibition of glycosylation fully rescued T-cell recognition of MSI tumors treated with MSS CM, restoring recognition to control levels. Critically, glycosylation blockade also significantly enhanced T-cell activation against native MSS PDOs, restoring it to levels comparable with those observed with MSI targets, implicating glycosylation-dependent immune evasion as an intrinsic feature of MSS tumor cells themselves. These results suggest that tumor-secreted factors can modulate the tumor-cell surface *in trans* through glycan remodeling, impairing the formation of a productive immune synapse and ultimately weakening immune recognition. Importantly, this effect operates primarily at the tumor-cell surface rather than through direct modulation of T-cell function. Siglec receptors, lectin-type receptors known to dampen immune responses upon recognition of sialylated glycans (29), were virtually absent on TCR-transduced T cells and were not induced by glycosylation inhibitor exposure. Consistently, pharmacologic glycosylation blockade at the concentrations used in coculture assays left T-cell viability, proliferation, and activation capacity fully intact. The glycosylation-dependent immune evasion we describe is therefore a tumor-intrinsic phenomenon, acting at the interface between cancer cells and T cells. Although pharmacologic inhibition proved effective, we also explored enzymatic deglycosylation using PNGase F as a complementary approach. Although PNGase F efficiently removed glycans from denatured protein extracts, it failed to act on intact viable PDOs. This is consistent with the known biochemical requirements of glycan-remodeling enzymes, which depend on protein denaturation to access glycosylation sites, rendering them inherently incompatible with live-cell systems in which proteins retain their native conformation. This underscores the value of pharmacologic inhibitors as the most suitable strategy to interrogate glycosylation-dependent mechanisms in intact live model systems.

The identity of the upstream secreted mediator(s) responsible for triggering glycan remodeling remains an open and important question. Targeted secretome profiling of MSS PDOs using a 96-plex immuno-oncology panel did not converge on a single dominant factor, pointing instead toward a combinatorial or noncanonical signal*,* potentially involving extracellular vesicles, metabolites, or lipid mediators*,* that falls outside the scope of antibody-based panels. In line with this, analysis of publicly available colorectal cancer RNA sequencing datasets revealed no coordinated transcriptional upregulation of glycosylation pathways in MSS versus MSI tumors, suggesting that the observed surface remodeling reflects posttranscriptional or posttranslational regulation rather than a hardwired transcriptional program. Dissecting these upstream mechanisms will be a key priority for future work.

It is therefore conceivable that, beyond lacking the high neoantigen load characteristic of MMRd tumors, MSS tumors may actively shape an immunosuppressive secretome that impairs immune recognition. Our findings highlight the tumor secretome as a critical modulator of immune responsiveness and suggest that targeting secreted factors from MSS tumors may provide new opportunities to overcome resistance in MSS cancers. In addition to informing new therapeutic strategies, this work provides a rationale for integrating secretome profiling into clinical decision-making. By identifying secretome-driven mechanisms that influence immune recognition, we shed light on new strategies for patient stratification, enabling the design of combination treatments tailored to the tumor's immunomodulatory profile. Such an approach could help extend the benefits of immunotherapy to patients with MSS tumors, who currently show limited responses to ICIs.

Importantly, although our study implicates glycosylation-dependent changes as key contributors to immune evasion, these mechanisms are unlikely to be exclusive. Other intrinsic pathways may similarly mediate resistance to T-cell pressure in MSS tumors, despite the presence of targetable antigens. This highlights a central insight of our work: MSS tumors possess an active, biologically encoded resistance to immune attack, beyond antigenicity alone. Crucially, our experimental strategy provides a framework to uncover such intrinsic immune escape mechanisms, offering a broadly applicable approach to dissecting tumor immunoresistance across cancer types and not limited to the MSS/MSI colorectal cancer context.

In conclusion, our data provide new transforming knowledge on how tumor cells interact and modulate the immune system of the host. Combinatorial treatments, designed to elevate neoantigen loads while at the same time neutralizing tumors’ intrinsic resistance, should be explored to foster immune surveillance of colorectal cancer tumors which are currently not eligible for immunotherapeutic treatment.

## Methods

### Human Subjects

Tumor tissue was collected from patients ages 18 years or older with stage I, II, III, or IV resectable colorectal cancer treated at Grande Ospedale Metropolitano Niguarda, Istituto Nazionale dei Tumori, Istituto Europeo di Oncologia, in Milan, AUSL della Romagna in Ravenna, and Ospedale di Santa Maria della Misericordia in Perugia. All patients provided written informed consent in accordance with the Declaration of Helsinki. The study was conducted under the ALFAOMEGA Master Observational Trial (NCT04120935), sponsored by Istituto Fondazione di Oncologia Molecolare ETS—The AIRC Institute of Molecular Oncology and approved by the Ethical Committee of each participating center. Tumor tissue for MSI_05 and MSI_06 was collected at the Netherlands Cancer Institute. The study (NL48824.031.14) was approved by the Medical Ethical Committee of the Netherlands Cancer Institute – Antoni van Leeuwenhoek hospital, and written informed consent was obtained from both patients.

### Organoid Culture

Tumor tissue was obtained by surgical resection and processed within 24 hours. Colorectal cancer organoids were established essentially as described in Cattaneo C.M. and colleagues, 2020 and Dijkstra and colleagues (22, 23). Briefly, tumor tissue was cut into small pieces and further processed using the Tumor Dissociation Kit (Miltenyi Biotec, cat. # 130-095-929) before embedding in Cultrex Reduced Growth Factor Basal Membrane Extract type 2 (RGF-BME-2, R&D Systems, cat. #3533-005-02). Colorectal cancer organoid medium is composed of Ad-DF (Advanced DMEM/F12; Gibco, cat #12634010), supplemented with 2 mmol/L GlutaMAX Supplement (Gibco, cat. #35050061), 10 mmol/L HEPES (Euroclone, cat. #ECM0180L), and 100/100 µ/mL penicillin/streptomycin (Euroclone, cat. #ECB3001L), 10% Noggin-conditioned medium, 20% R-spondin1–conditioned medium, 1× B27 supplement without vitamin A (Gibco, cat. #12587010), 1.25 nmol/L N-acetylcysteine (Sigma-Aldrich, cat. #A9165), 10 mmol/L nicotinamide (Sigma-Aldrich, cat. #N0636), 50 ng/mL human recombinant EGF (PeproTech, cat. #AF-100-15), 500 nmol/L A83-01 (Tocris Bioscience, cat. #2939), 3 µmol/L SB202190 (Sigma-Aldrich, cat. #S7067), and 10 nmol/L prostaglandin E2 (Cayman Chemicals, cat. #14010-1). Organoids were passaged approximately every 1 to 2 weeks by incubating in TrypLE Express (Gibco, cat. #12605028) for 5 to 10 minutes at 37°C to dissociate organoids to single cells and replating in fresh BME. After passaging, 10 µmol/L Y-27632 (Selleck Chemicals, cat. #S1049) was added to colorectal cancer medium for the first 2 to 3 days. Organoids were cryopreserved in 10% DMSO/FBS (Euroclone, cat. #EMR385250; Sigma-Aldrich, cat. #F7524). Organoids were regularly checked for *Mycoplasma* contamination. The identity of each cell line was checked before starting each experiment and after every genomic DNA extraction by GenePrint 10 system (Promega, cat. #B9510) through short tandem repeats at 16 different loci.

### IHC

Organoids were recovered from BME, pelleted, and fixed in 4% paraformaldehyde for 2 hours at room temperature. Samples were embedded in paraffin blocks using Diapath automatic processor. Sections of approximately 4 µm were cut and counterstained with hematoxylin and eosin (Diapath) according to standard protocol. Samples were mounted in Eukitt (Bio Optica) for histopathologic analysis. For assessment of MMR status, IHC was performed according to standard protocols. Paraffin was removed with xylene, and the sections were rehydrated in graded alcohol. Antigen retrieval was carried out using preheated target retrieval solution for 30 minutes or using 10 mmol/L citrate buffer (anti-MSH2) or Tris-EDTA buffer pH 9.0 (anti-MLH1, anti-MSH6, and anti-PMS2) heated for 30 minutes and blocked, and endogenous peroxidase was quenched with 3% hydrogen peroxide in distilled water for 10 minutes at room temperature. Tissue sections were blocked with FBS serum in PBS for 60 minutes and incubated 2 hours with the following primary antibodies, diluted 1:30: MLH1, clone G168-15 (Biocare Medical, cat. #PM 220 AA, RRID: AB_10581025); MSH2, clone FE11 (Biocare Medical, cat. #PM 219 AA, RRID: AB_10581790); MSH6, clone 44 (Biocare Medical, cat. #PM265 AA, RRID: AB_10581791); and PMS2, clone A16-4 (Biocare Medical, cat. #PM344 AA, RRID: AB_10581487). The antibody binding was detected using a polymer detection kit (GAM-HRP, Microtech) followed by a diaminobenzidine (DAB) chromogen reaction (peroxidase substrate kit, DAB, SK-4100; Vector Labs). All sections were counterstained with Mayer’s hematoxylin and visualized using a BrightField microscope (Leica DM750). Samples were analyzed for the absence or presence of nuclear staining of the MMR proteins. The IHC results were confirmed by a pathologist who was blinded to the results.

### Organoid Surface Staining

Organoids were dissociated to single cells using TrypLE Express, with or without 24-hour preincubation with 200 ng/mL IFNγ (PeproTech, cat. #300-02). Tumor cells were washed in FACS buffer (PBS supplemented with 5 mmol/L EDTA and 1% BSA) and stained using the following antibodies: mouse anti-human HLA-A,B,C-PE (BD Biosciences, cat. #555553, RRID: AB_395936; clone G46-2.6, used 1:50), mouse anti-human HLA-A2-APC (BD Biosciences, cat. #561341, RRID: AB_10646036; clone BB7.2, used 1:50), mouse anti-human HLA-DP/DQ/DR-FITC (BD Biosciences, cat. #550853, RRID: AB_393926; clone Tü39, used 1:50), and mouse anti-human CD274-APC (eBioscience, cat. #17-5983-41, RRID: AB_10597280; clone MIH1, used 1:200). Antibodies were used according to the manufacturer’s instruction. To exclude dead cells, we used labeling with near-IR viability dye (Invitrogen, cat. #L10119), Zombie Aqua Fixable Viability Kit (BioLegend, cat. #423101), or 4′,6-diamidino-2-phenylindole (DAPI), following the manufacturer’s instruction. Cells were recorded using a Canto II flow cytometer or CytoFLEX S flow cytometer.

### T-cell Engineering and Culture

Healthy donor–derived T cells are engineered as described in Cattaneo and colleagues (21). Briefly, TCR α and β variable sequences of the selected TCR were synthesized and subcloned into a modified pMP71 retroviral vector that contains mouse TCR constant regions and the puromycin N-acetyltransferase resistance gene. Retrovirus was produced by transfecting FLY-RD18 packaging cells (RRID: CVCL_8871) with pMP71-TCR plasmid DNA using X-tremeGENE 9 transfection reagent (Merck Millipore, cat. # 6365779001). In the meantime, CD8^+^ T cells were isolated from healthy donor PBMCs using the CD8^+^ T Cell Isolation Kit (Miltenyi Biotec, cat. #130-096-495) and stimulated with 5 µg/mL plate-bound aCD3/CD28 (eBioscience, cat. #16-0037-85, clone OKT-3, RRID: AB_468855; eBioscience, cat. #16-0289-81, clone CD28.2, RRID: AB_468926) in T-cell medium [composed of RPMI 1640 medium (Euroclone, cat. #ECB9006L)], supplemented with 10% human AB serum (Euroclone, cat. #ECS0219D), 100/100 µ/mL penicillin/streptomycin, 2 mmol/L UltraGlutamine I, and 150 U/mL human recombinant IL2 (R&D Systems, cat. #200-IL). After 48 hours, retroviral supernatants were collected and used to infect prestimulated CD8^+^ T cells by spinoculation (2,000 *g* for 90 minutes) in RetroNectin-coated (Takara, cat. #T100B) plates. Transduction efficiency was measured 72  hours later by staining with anti-mouse TCRβ-PE (BD Biosciences, cat. #561081; clone H57-597, RRID: AB_10563767, used 1:150) and analyzed by flow cytometry. TCR-transduced T cells were then selected with 2.5  µg/mL puromycin (Vinci-Adipogen, cat. #AG-CN2-0078-M100). Then, transduced T cells were expanded using the rapid expansion protocol (REP) with a 1:1 mixture of RPMI 1640 and AIM-V medium (Gibco, cat. #12055091) supplemented with 5% human AB serum, 30  ng/mL anti-CD3 antibody, and 3,000 U/mL human recombinant IL2 in the presence of irradiated (40 Gy) allogeneic PBMCs (200:1 feeder/T cell ratio). After 7 days of REP culture, the medium was refreshed with new IL2 every 2 days for a total REP culture time of 14 days. After REP, transduced T cells were cryopreserved in 10% DMSO/FBS or kept in culture at ∼2e^6^ cells/mL in T-cell medium.

### Tumor Recognition Assays

Tumor organoids were previously stimulated with 200 ng/mL IFNγ (PeproTech, cat. #300-02) for 24 hours. After dissociation into single cells using TrypLE Express (Gibco, cat. #12605028), organoids were resuspended in PBS containing varying concentrations of MART-1 peptide (ELAGIGILTV or EAAGIGILTV; 0/0.02/0.1/0.5 µmol/L) and incubated for 30 minutes at 37°C. Organoids were then washed twice with PBS and resuspended in T-cell medium. For glycosylation-blocking experiments, tumor organoids were pretreated after dissociation with 2.5 µmol/L Benzyl-α-GalNAc (MedChemExpress, cat. #HY-129389) and 100 µmol/L lithocholic acid O-aspartate (Lith-O-Asp; MedChemExpress, cat. #HY-112415) for 2 hours at 37°C. Following washes, these organoids were pulsed with 0.02 µmol/L MART-1_ELA_ peptide for 30 minutes at 37°C before final washing and resuspension in T-cell medium.

TCR-transduced CD8^+^ T cells were seeded with tumor organoids at a 1:2 T cell-to-organoid ratio in anti-CD28–coated (eBioscience, cat. #16-0289-81, clone CD28.2, RRID: AB_468926) U-bottom 96-well plates, cultured in either standard T-cell medium or CM as specified. For glycosylation inhibition assays, media were supplemented with 0.25 µmol/L benzyl-α-GalNAc and 10 µmol/L Lith-O-Asp throughout coculture. When applicable, 20 µg/mL anti–PD-1 blocking antibody (nivolumab) was added. T cells cultured without target cells served as negative controls, whereas stimulation with 50 ng/mL phorbol 12-myristate 13-acetate (Sigma-Aldrich, cat. #19-144) and 1 µg/mL ionomycin (Sigma-Aldrich, cat. #I9657) functioned as positive controls.

For CD137 surface expression, after 24-hour incubation with tumor organoids, T cells were washed twice in FACS buffer and stained with anti-human CD3–PerCP-Cy5.5 (BD Biosciences, cat. #332771, RRID: AB_2868620, clone SK7, 1:20), anti-human CD8-BV421 (BD Biosciences, cat. #562429, RRID: AB_11153676, clone RPA-T8, 1:200), anti-mouse TCRβ–PE (BD Biosciences, cat. #561081, RRID: AB_10563767, clone H57-597, 1:150), anti-human CD137–APC (BD Biosciences, cat. #550890, RRID: AB_398477, clone 4B4-1, 1:30), and near-IR viability dye (Invitrogen, cat. #L10119) for 30 minutes at 4°C in the dark. Cells were washed and acquired on a Canto II flow cytometer. For intracellular IFNγ analysis, after 1-hour coculture with tumor organoids, Golgi-Plug (BD Biosciences, cat. #555029) and Golgi-Stop (BD Biosciences, cat. #554724) were added, and incubation continued for 4 hours. Cells were washed twice; stained with anti-human CD3–PerCP-Cy5.5, anti-human CD8-BV421, anti-mouse TCRβ–PE, and viability dye; fixed; permeabilized; and stained intracellularly for IFNγ (BD Biosciences, cat #554702, RRID: AB_398580, clone B27, 1:50) using the Cytofix/Cytoperm kit (BD Biosciences, cat. #554714, RRID: AB_2869008). Cells were then resuspended in FACS buffer and recorded using a Canto II flow cytometer.

### Tumor Killing Assay

PDOs were previously stimulated with 200 ng/mL IFNγ (PeproTech, cat. #300-02) for 24 hours. Then, after dissociation into single cells using TrypLE Express (Gibco, cat. #12605028), organoids were stained with 100 nmol/L CellTrace Far Red (Invitrogen, cat. #C34564) in PBS for 20 minutes at 37°C, and subsequently washed twice with 10% FBS/PBS. Next, tumor organoids were resuspended in PBS in the presence of 0 or 0.02 µmol/L of MART-1 peptide (ELAGIGILTV). After 30-minute incubation at 37°C, organoids were washed twice in PBS and resuspended in T-cell medium.

Tumor organoids were then seeded alone or with the TCR-transduced CD8^+^ T cells (5:1 T cell/organoid ratio) in anti-CD28–coated (eBioscience, cat. #16-0289-81, clone CD28.2, RRID: AB_468926) U-bottom 96-well plates in T-cell medium or CM (collected as described in the “CM Collection” section below) supplemented with 20 µg/mL anti–PD-1 blocking antibody. After 24-hour incubation, pictures of each well were taken using a fluorescence microscope, for the quantification of the Far Red signal, using FIJI. Additionally, a flow cytometry–based quantification was performed. Organoids and T cells were collected and dissociated into single cells with TrypLE Express. AccuCount blank counting beads (7.46 µm; Spherotech, cat. #ACBP-70-10) were added to each well in equal number. Cells were then washed twice in FACS buffer and stained with DAPI and recorded using a Canto II flow cytometer.

### Cytokine, Chemokine, and Immunomodulatory Molecule Release Assays

For the analysis of cytokine, supernatants of the CD137-mediated recognition assay were collected after 24 hours in culture. The supernatants were immediately frozen and preserved at −80°C. The supernatants were thawed on ice, and the presence of the indicated cytokines was detected using the Cytometric Beads Array Human IFNγ Flex Set (BD Biosciences, cat. #558269, RRID: AB_2869127) or the LEGENDplex Human CD8/NK Panel (BioLegend, cat. #741065) according to the manufacturers’ instructions. For the analysis of chemokines, CM of the different organoids was collect as described in the “CM Collection” section below and used as supernatant. Supernatants were used fresh or immediately frozen, preserved at −80°C, and thawed on ice when needed. The presence of the indicated chemokines was detected using the LEGENDplex Human Proinflammatory Chemokine Panel 1 (BioLegend, cat. #741081) and Olink Immuno-Oncology Target 96 Kit according to the manufacturers’ instructions.

### CM Collection

Tumor organoids were previously stimulated with 200 ng/mL IFNγ (PeproTech, cat. #300-02) for 24 hours. Then, after dissociation into single cells using TrypLE Express, organoids were seeded in fresh T-cell medium at 2.5e^5^ cells/mL. Twenty-four hours later, the supernatant was collected and spun at 1,500 rpm for 5 minutes to remove any debris. The supernatant was used fresh or immediately frozen and preserved at −80°C.

### T-cell Viability, Functionality, and Proliferation Assays

To determine cell viability, TCR-transduced CD8^+^ T cells were seeded at a concentration of 1 × 10^6^ cells/mL in anti-CD28–coated (eBioscience, cat. #16-0289-81, clone CD28.2, RRID: AB_468926) U-bottom 96-well plates in T-cell medium or CM (collected as described). CellTiter-Glo Luminescent Cell Viability Assay (Promega, cat. #G7573) was used at the time of seeding and again after 24 hours. Luminescence was measured using EnVision (PerkinElmer).

Activation of T cells was evaluated by intracellular IFNγ expression. Briefly, HLA-A2^+^ immortalized B cells (20) were incubated with 0 or 0.02 µmol/L of MART-1_ELA_ peptide for 30 minutes at 37°C, followed by two washed in PBS. Subsequently, B cells were resuspended in T-cell medium or CM. TCR-transduced CD8^+^ T cells were then seeded with B cells (1:2 T cell/B cell ratio) in anti-CD28–coated U-bottom 96-well plates using T-cell medium or CM (collected as described). Golgi-Plug (BD Biosciences, cat. #555029) and Golgi-Stop (BD Biosciences, cat. #554724) were added to the coculture and incubated for an additional 4 hours. Following coculture, cells were washed twice in FACS buffer and stained with anti-human CD3-PerCP-Cy5.5 (BD Biosciences, cat. #332771, RRID: AB_2868620, clone SK7, 1:20), anti-human CD8-BV421 (BD Biosciences, cat. #562429, RRID: AB_11153676, clone RPA-T8, 1:200), anti-mouse TCRβ-PE (BD Biosciences, cat. #561081, RRID: AB_10563767, clone H57-597, 1:150), and near-IR viability dye (Invitrogen, cat. #L10119) for 30 minutes at 4°C. Cells were washed twice in FACS buffer, fixed, and stained for intracellular IFNγ (anti-human IFNγ-APC, (BD Biosciences, cat. #554702, RRID: AB_398580, clone B27, used 1:50) using the Cytofix/Cytoperm kit (BD Biosciences, cat. #554714, RRID: AB_2869008) according to the manufacturer’s instructions. Cells were then resuspended in FACS buffer and recorded using a Canto II flow cytometer.

To determine T-cell proliferation, TCR-transduced T cells were labeled with 100 nmol/L CellTrace Far Red (Invitrogen, cat. #C34564) following the manufacturer’s instruction. Then, T cells were seeded at a concentration of 1 × 10^6^ cells/mL in anti-CD28–coated U-bottom 96-well plates, with or without MART-1_ELA_ -loaded B cells (2:1 T cells/B-cell ratio), for stimulated and unstimulated conditions, respectively. The assay was performed in T-cell media or in MMRp-derived CM. After 72 hours, cells were resuspended in FACS buffer, stained with DAPI, and recorded using a CytoFLEX S flow cytometer.

### T-cell Engagement Assay

To quantify physical engagement between T cells and tumor cells, we used a high-content imaging assay based on cocultures of fluorescently labeled T cells and tumor organoids. Two days prior to the assay, PDOs were enzymatically dissociated into single cells and passed through a 40-μm cell strainer to ensure uniform organoid size. Organoids were re-aggregated and maintained in culture for 24 hours and then stimulated with 200 ng/mL IFNγ for an additional 24 hours to enhance antigen presentation. On the day of the assay, organoids were labeled with Cytolight Red (Sartorius, cat. #4705) according to the manufacturer’s instructions and pulsed with 0.02 µmol/L MART-1_ELA_ peptide for 30 minutes at 37°C. Organoids were seeded in flat-bottom 96-well plates at 1,000 organoids per well, corresponding to approximately 10,000 single-cell equivalents. T cells were labeled using CellTrace CFSE Cell Proliferation Kit (Invitrogen, cat. #C34554) following the manufacturer’s protocol and added to the wells at an effector-to-target ratio of 1:2. Cocultures were maintained in either standard T-cell medium or tumor-conditioned medium (collected as described earlier), supplemented with 20 μg/mL anti–PD-L1 blocking antibody (nivolumab). Plates were imaged after 48 hours using the Incucyte Live-Cell Analysis System. T-cell engagement was quantified using FIJI by measuring the proportion of T cells in direct contact with tumor organoids. Engaged T cells were expressed as a percentage of total segmented T cells per field of view.

### T-cell Avidity Assay

The binding strength between T cells and tumor cells was assessed using the z-Movi Cell Avidity Analyzer (LUMICKS) according to the manufacturer’s protocol with custom adaptations. Tumor organoids were first stimulated with 200 ng/mL IFNγ for 24 hours and then enzymatically dissociated into single cells. Tumor cells were seeded onto z-Movi glass-bottom flow chambers at a density of 1.5 × 10^8^ cells/mL to form a confluent monolayer. Depending on the experiment, the glass surface was precoated with either poly-L-lysine (Sigma-Aldrich, cat. #P4707) or concanavalin A (Sigma-Aldrich, cat. #C2010). Peptide loading was performed directly on the adhered tumor cells using 100 µmol/L MART-1_ELA_ peptide for 1 hour at 37°C. For experiments involving CM, tumor cells were preincubated with the indicated CM for 24 hours prior to the assay, and the same CM was used during the measurement.

Effector T cells were labeled with CellTrace Far Red (Invitrogen, cat. #C34564) and resuspended at 1 × 10^7^ cells/mL. During the assay, individual T cells in suspension were acoustically positioned onto the tumor monolayer. The contact time between T cells and tumor cells was set to 10 minutes (docking phase), after which a gradually increasing acoustic force was applied to detach the T cells.

The output was represented as the percentage of bound T cells as a function of the applied force, generating binding strength curves for each condition. Data were acquired and processed using the LUMICKS software suite.

### Membrane Proteome after Exposure to CM

MSI tumor organoids were previously stimulated with 200 ng/mL IFNγ for 24 hours, as performed in the functional assays. Then, organoids were dissociated into single cells using TrypLE Express and resuspended in CM (collected as described previously). Cells were seeded in a 24-well plate and incubated for 24 hours at 37°C. After incubation, tumor cells were collected, washed in PBS, and processed for membrane protein extraction using the Mem-PER Plus Membrane Protein Extraction Kit (Thermo Fisher Scientific, cat. #89842), following the manufacturer’s instructions. Membrane proteome was quantified using the Pierce BCA Protein Assay Kit (Thermo Fisher Scientific, cat. #23225). Then, the membrane protein fraction was digested for LC-MS/MS analysis. Briefly, 25 μg of membrane fraction were resuspended in 200 μL of UA buffer (100 mmol/L Tris HCl pH 8.0, 8 mol/L urea) and transferred into microcon filters with 10 kDa cutoff (Millipore). After four washes with 300 μL UA buffer, cysteine reduction and alkylation were performed adding 10 mmol/L TCEP (Thermo Fisher Scientific) and 40 mmol/L 2-chloroacetamide (Sigma-Aldrich) in UA buffer for 30 minutes at room temperature. After two washes with UA buffer and one with 100 mmol/L Tris HCl, pH 8.0, protein in-solution digestion was performed by incubating each sample in 100 μL of 100 mmol/L Tris HCl, pH 8.0, supplemented with 1 μg of trypsin overnight at 37°C. Then, an additional incubation with 1 μg of trypsin for 3 hours was performed. The obtained peptides were collected by centrifugation and purified on a C18 StageTip.

### Global and Membrane Proteomics MS Analysis

A volume of 1.5 μL of digested sample was injected onto an Exploris 480 mass spectrometer (Thermo Fisher Scientific) equipped with FAIMS device (Thermo Fisher Scientific). Columns were packed in-house with ReproSil-Pur C18-AQ beads (Dr. Maisch Gmbh), 1.9 μm of diameter, using a high-pressure bomb loader (Proxeon).

Peptides separation was achieved with a linear gradient from 95% solvent A (2% ACN and 0.1% formic acid) to 41% solvent B (80% ACN and 0.1% formic acid) over 124 minutes and from 41% to 100% solvent B in 9 minutes at a constant flow rate of 0.25 μL/minutes, with a single run time of 131 minutes. The mass spectrometer was operated in data-independent acquisition (DIA) mode with the following parameters: charge state: 2 to 6, MS1 resolution = 120,000, precursor range = 400 to 1,000 m/z, MS1 automatic gain control target = 3 × 106 (300%), MS1 maximum injection time = 55 milliseconds, MS2 resolution = 30,000, MS2 automatic gain control target = 10 × 106 (1000%), MS2 maximum fill time = auto, radio frequency lens 40%, and MS2 HCD collision energy % = 32, 30 m/z isolation window. The MS2 scan range was auto, and the loop control was set to “all.”

The FAIMS device was operated with voltages −50  and −70 V, with a total carrier gas flow of 3.7  L/minutes at 300°C for ion transfer tube temperature.

### DIA MS Analysis and Database Search

The DIA raw files of FAIMS-DIA were searched by Spectronaut version 18. These searches were done in direct DIA mode. The default BGS factory settings, using UniProt cp Human proteome reference, were used. For DIA searches comparison, trypsin was selected as the digestion enzyme. Carbamidomethylation was selected as a fixed modification, whereas methionine oxidation and N-terminal acetylation were selected as variable modifications. FDRs of PSMs and peptide/protein groups were set to 0.01. “Area” MS2 quantity type was used with cross-run normalization “ON”. Data are available via ProteomeXchange (37) with identifier PXD066343.

## Supplementary Material

Supplementary Table 1 Complete list of membrane proteins identified by mass spectrometry in MSI_03 organoids exposed to MSI- or MSS-conditioned media.

Supplementary Table 2 Statistically significant membrane proteins differentially expressed in MSI_03 organoids exposed to MSI- vs MSS-conditioned media.

Supplementary Table 3 Complete list of membrane proteins indentified by mass spectometry in MSI_06 organoids exposed to MSI- or MSS-conditioned media.

Supplementary Table 4 Statistically significant membrane proteins differentially expressed in MSI_06 organoids exposed to MSI- vs MSS-conditioned media.

Supplementary Table 5 Complete list of membrane proteins indentified by mass spectometry in MSI_05 organoids exposed to MSI- or MSS-conditioned media.

Supplementary Table 6 Statistically significant membrane proteins differentially expressed in MSI_05 organoids exposed to MSI- vs MSS-conditioned media.

Supplementary Figure 1 Immunohistochemical characterization of patient-derived colorectal cancer organoids, including H&E staining and MMR protein expression.

Supplementary Figure 2 Flow cytometry analysis of MHC class I, MHC class II, and HLA-A2 cell-surface expression in patient-derived organoids.

Supplementary Figure 3 Flow cytometry analysis of PD-L1 cell-surface expression in patient-derived organoids.

Supplementary Figure 4 Flow cytometry characterization of CD8+ T cells retrovirally transduced with a antigen-specific TCR and validation of antigen-specific reactivity.

Supplementary Figure 5 CD137 expression on T cells cocultured with MSS and MSI tumor organoids across increasing antigen concentrations.

Supplementary Figure 6 IFNγ release in supernatants from T cells cocultured with MSS and MSI tumor organoids across increasing antigen concentrations.

Supplementary Figure 7 TNFα, Granzyme-A, and Granzyme-B release in supernatants from T cells cocultured with MSS and MSI tumor organoids.

Supplementary Figure 8 IFNγ release and CD137 expression in T cells cocultured with MSS and MSI tumor organoids using a weakly immunogenic antigen.

Supplementary Figure 9 Heatmap of immunomodulatory molecule levels in MSS and MSI organoid supernatants.

Supplementary Figure 10 Effect of MSS and MSI organoid-derived conditioned media on T cell viability, proliferation, IFNγ production, and binding strength to tumor organoids, as well as on immunomodulatory molecule expression in MSI organoids.

Supplementary Figure 11 Pathway enrichment analysis of membrane proteins upregulated in MSI organoids treated with MSS-conditioned media, and overlap with glycosylation-related proteins.

Supplementary Figure 12 Intracellular IFNγ production in T cells cocultured with glycosylation inhibitor-treated MSI organoids in the presence of MSS-conditioned media.

## Data Availability

All data generated and/or analyzed during this study are included in this published article. Additional information can be requested to the corresponding authors. The proteomics data have been deposited in the ProteomeXchange Consortium via the PRIDE partner repository with the dataset identifier PXD066343.
