# Supplementary material for: Mismatch Repair–Proficient Colorectal Cancer Can Evade Immune Surveillance through an Intrinsic Suppressive Program
Source: Cancer Discov. 2026 May 13;16(10):1992–2007. doi: 10.1158/2159-8290.CD-26-0542 (PMC13628094; doi:10.1158/2159-8290.CD-26-0542)
Supplement: Supplementary file 1 [file cd-26-0542_supplementary_figure_10_suppsf10.pdf]

Supplementary figure 10.

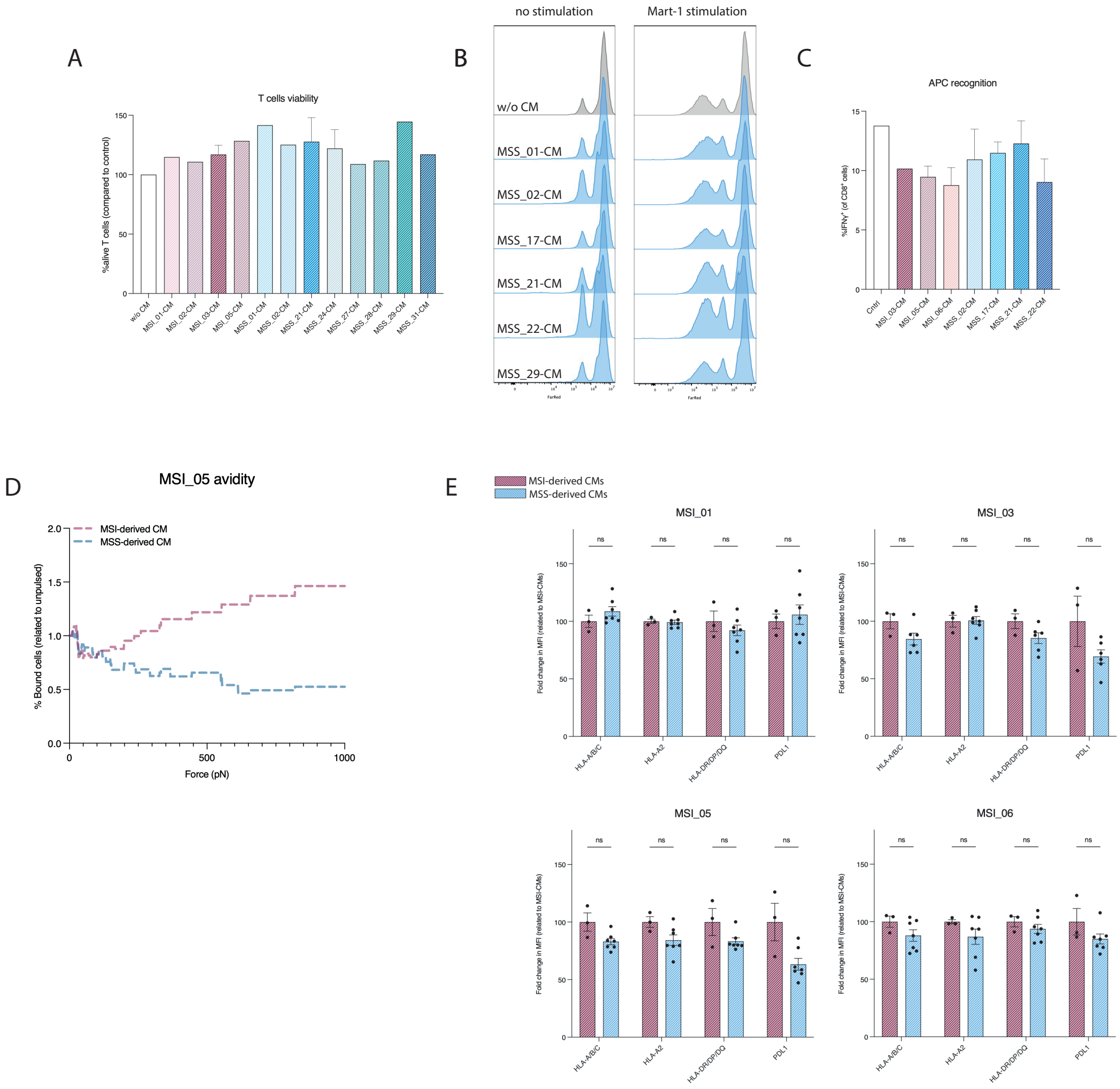

Supplementary figure 10. Analysis of T cell functionality and tumor cell phenotype upon exposure to tumor organoids-derived conditioned media

A) Quantification of T cell viability after 24 hours exposure to different PDOs-derived conditioned media. Error bars represent SEM of at least two biological replicates. *$p<0.05$; **$p <0.01$; ***$p<0.001$; ****$p<0.0001$; ns $p>0.05$ (not shown on the graph); Ordinary one-way ANOVA, multiple comparisons.

B) Proliferation rate of T cells. Histograms represent the intensity of the FarRed fluorescence in stimulated (right) or unstimulated (left) T cells after 72 hours of culture. Peaks indicated the extent of T cells growth.

C) Quantification of APC induced IFNγ intracellular expression, in the presence of the indicated conditioned media. The background (spontaneous IFNγ expression) was subtracted from the signal. Error bars represent SEM of at least two biological replicates. *$p<0.05$; **$p <0.01$; ***$p<0.001$; ****$p<0.0001$; ns $p>0.05$ (not shown on the graph); Ordinary one-way ANOVA, multiple comparisons.

D) Single-cell force spectroscopy assay assessing the effect of conditioned media on T cell binding strength to an MMSI tumor organoid. A single MSI organoids was pre-incubated for 24 hours with CM derived from either MSI (pink) or MSS (blue) tumors. The binding assay was performed in the continued presence of the same CM used for pre-treatment. Data were normalized to the unpulsed control of each condition. Mean ± SEM of biological replicates are shown. Binding strength is expressed as the percentage of T cells remaining attached under increasing acoustic force.

E) Cell-surface expression of immunomodulatory molecules in patient derived MSI organoids as determined by flow cytometry. Organoids were stimulated with 200 ng/mL IFNγ and kept 24 hours in the presence of MSS- (showed in blue) or MSI- (showed in purple) derived conditioned media. Data are normalized on the changes induced by MSI-derived conditioned media. Black dots represent different patient-derived organoid conditioned media.
