# Supplementary material for: Mismatch Repair–Proficient Colorectal Cancer Can Evade Immune Surveillance through an Intrinsic Suppressive Program
Source: Cancer Discov. 2026 May 13;16(10):1992–2007. doi: 10.1158/2159-8290.CD-26-0542 (PMC13628094; doi:10.1158/2159-8290.CD-26-0542)
Supplement: Supplementary file 2 [file cd-26-0542_supplementary_figure_11_suppsf11.pdf]

Supplementary Figure 11.

A

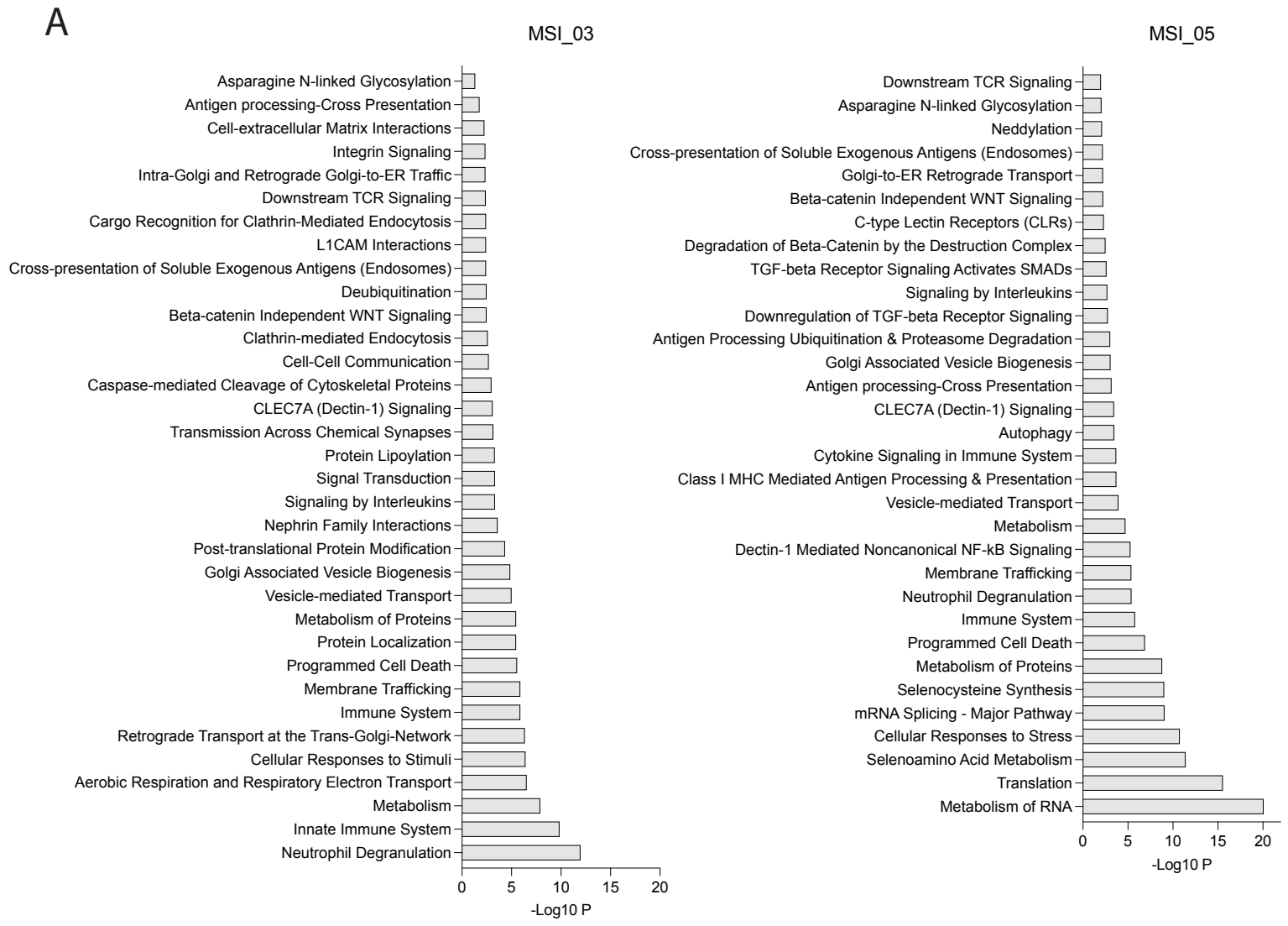

B

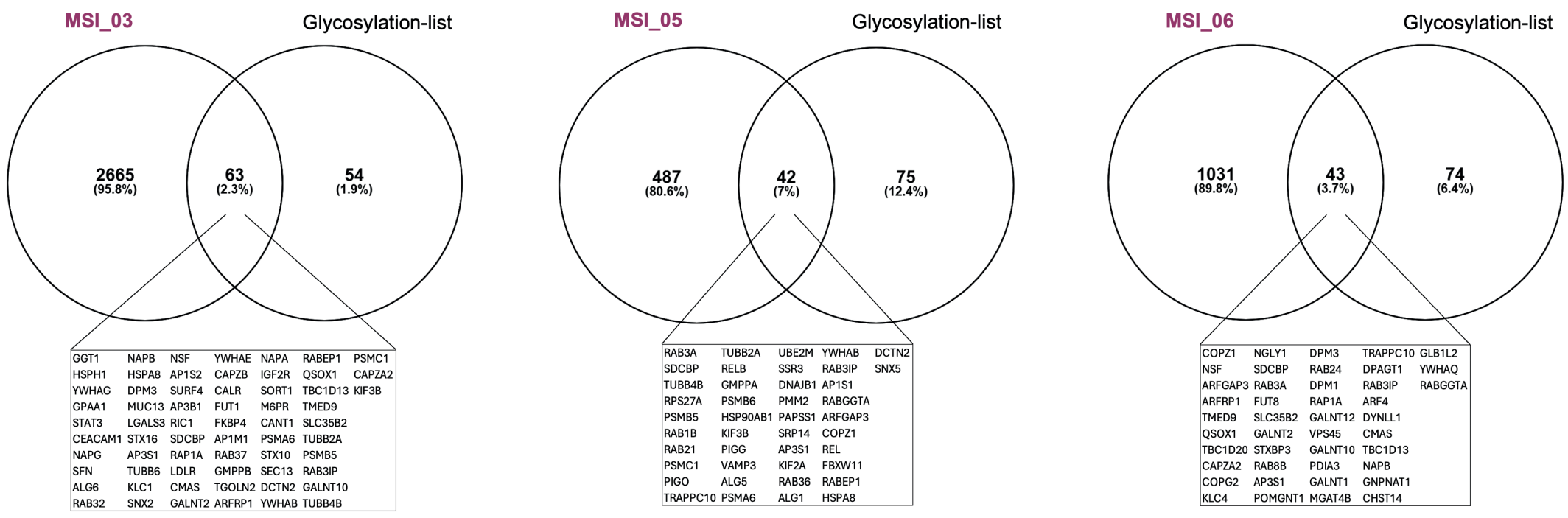

Supplementary Figure 11. Pathway analysis of membrane proteins in MSI tumor cells exposed to MSI- or MSS-derived conditioned media.

A) Annotations of proteins significantly upregulated in the different MSI tumor organoids treated with MSS-conditioned media. Reactome pathways enrichment analysis performed by the web tool EnrichR. Pathways categories and their relative average of enrichment scores (average -log10 p-value) were plotted as histogram. Pathway directly or indirectly linked to glycosylation are shown in black and grey, respectively.

B) Identification of glycosylation-related membrane proteins modulated by conditioned media.

Venn diagrams showing the overlap between significantly regulated membrane proteins identified by mass spectrometry in MSI colorectal cancer organoids exposed to MSI- or MSS-conditioned media (displayed in the hitmaps from Figure 3) and proteins associated with glycosylation-related pathways identified through the pathway enrichment analysis performed across all samples. Percentages indicate the fraction of overlapping proteins relative to the total number of significantly modulated membrane proteins in each PDO. Proteins included in the overlap are listed in the tables below each diagram.
