# Supplementary material for: Mismatch Repair–Proficient Colorectal Cancer Can Evade Immune Surveillance through an Intrinsic Suppressive Program
Source: Cancer Discov. 2026 May 13;16(10):1992–2007. doi: 10.1158/2159-8290.CD-26-0542 (PMC13628094; doi:10.1158/2159-8290.CD-26-0542)
Supplement: Supplementary file 3 [file cd-26-0542_supplementary_figure_12_suppsf12.pdf]

Supplementary Figure 12.

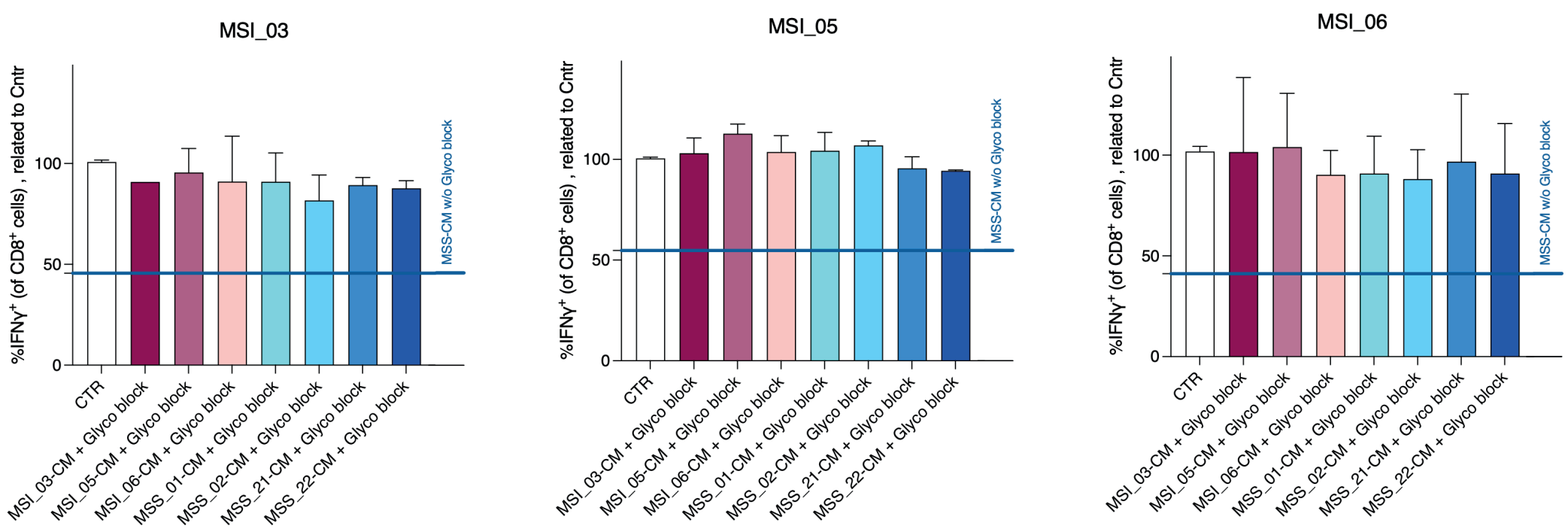

Supplementary Figure 12. Effect of glycosylation blockade on MSS-conditioned media–mediated suppression of T cell IFNγ production.

Quantification of intracellular IFNγ expression in T cells co-cultured with MSI tumor organoids pre-treated with glycosylation inhibitors, in the presence of conditioned media with glycosylation blocking. The blue line represents the average suppressive effect of MSS-conditioned media without glycosylation inhibition. The control condition (no conditioned media) was used as reference for normalization. Background signal (spontaneous IFNγ expression) was subtracted. Error bars indicate SEM from at least two biological replicates.
