# Supplementary material for: Mismatch Repair–Proficient Colorectal Cancer Can Evade Immune Surveillance through an Intrinsic Suppressive Program
Source: Cancer Discov. 2026 May 13;16(10):1992–2007. doi: 10.1158/2159-8290.CD-26-0542 (PMC13628094; doi:10.1158/2159-8290.CD-26-0542)
Supplement: Supplementary file 4 [file cd-26-0542_supplementary_figure_1_suppsf1.pdf]

Supplementary figure 1.

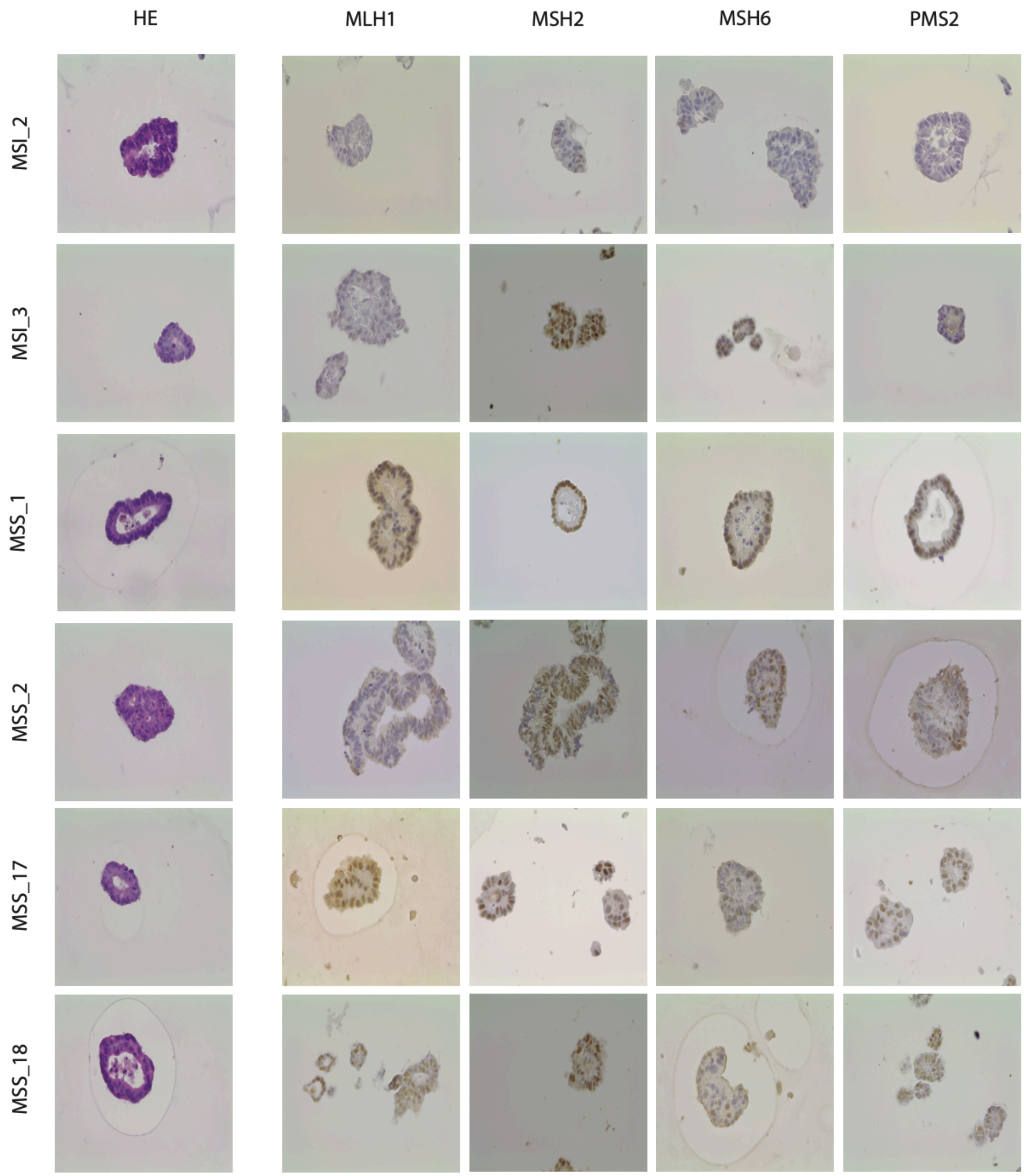

Supplementary figure 1. Immunohistochemistry characterization of patient-derived CRC tumor organoids. Patient derived tumor organoids were fixed, paraffin-embedded and sequential slides were stained with hematoxylin and eosin or antibodies against MLH1, MSH2, MSH6 and PMS2.
