# Supplementary material for: Mismatch Repair–Proficient Colorectal Cancer Can Evade Immune Surveillance through an Intrinsic Suppressive Program
Source: Cancer Discov. 2026 May 13;16(10):1992–2007. doi: 10.1158/2159-8290.CD-26-0542 (PMC13628094; doi:10.1158/2159-8290.CD-26-0542)
Supplement: Supplementary file 6 [file cd-26-0542_supplementary_figure_3_suppsf3.pdf]

Supplementary figure 3.

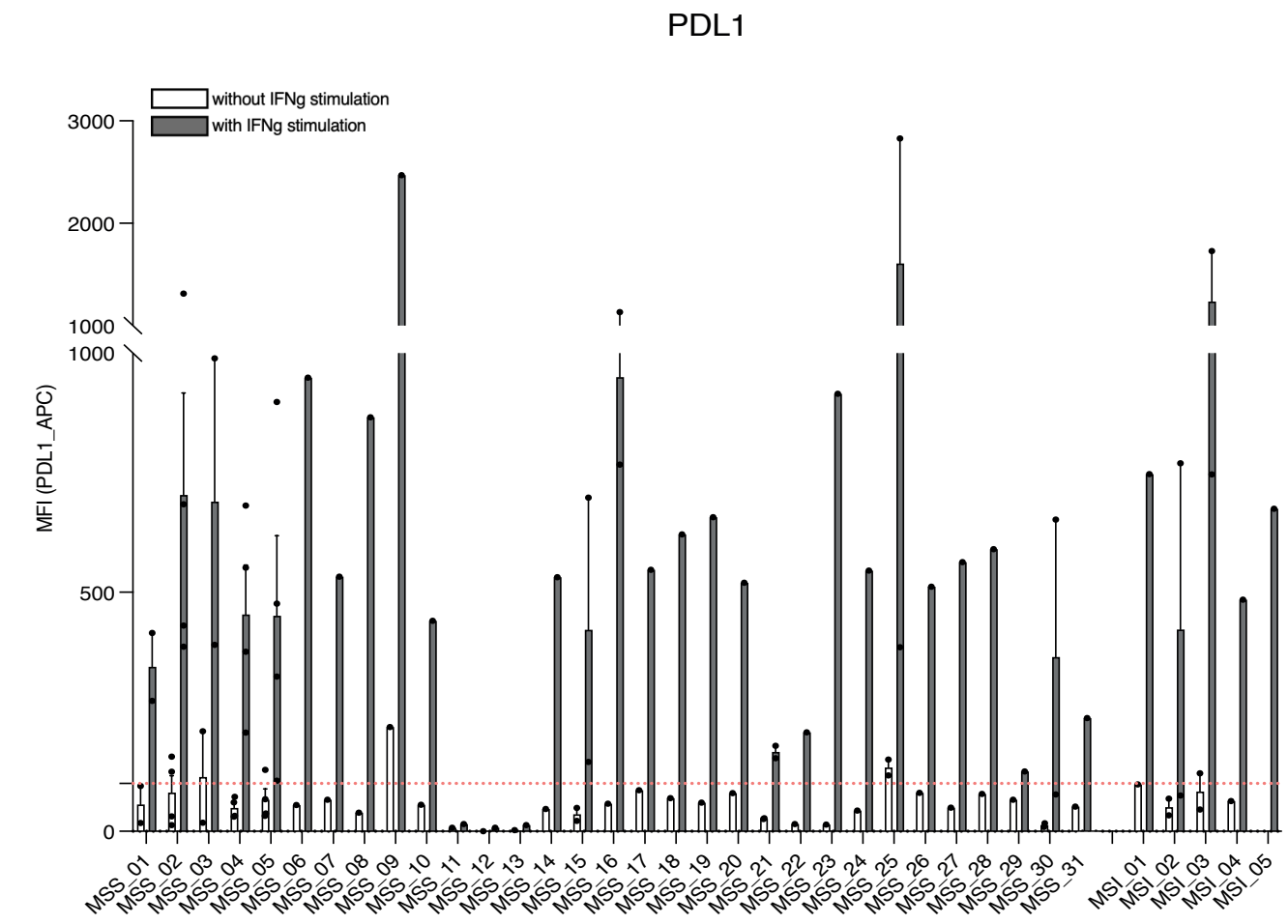

Supplementary figure 3. Cell-surface expression of PDL-1 molecule in PDOs.

Cell-surface expression of PDL-1 as determined by flow cytometry. Organoids were stimulated with 200 ng/mL IFNγ for 24 hours (grey bars) or left unstimulated (white bars). Bar graphs indicate median fluorescence intensity (MFI). Error bars represent SEM of at least two independent experiments. Dots indicate biological replicates.
