# Supplementary material for: Mismatch Repair–Proficient Colorectal Cancer Can Evade Immune Surveillance through an Intrinsic Suppressive Program
Source: Cancer Discov. 2026 May 13;16(10):1992–2007. doi: 10.1158/2159-8290.CD-26-0542 (PMC13628094; doi:10.1158/2159-8290.CD-26-0542)
Supplement: Supplementary file 7 [file cd-26-0542_supplementary_figure_4_suppsf4.pdf]

Supplementary figure 4.

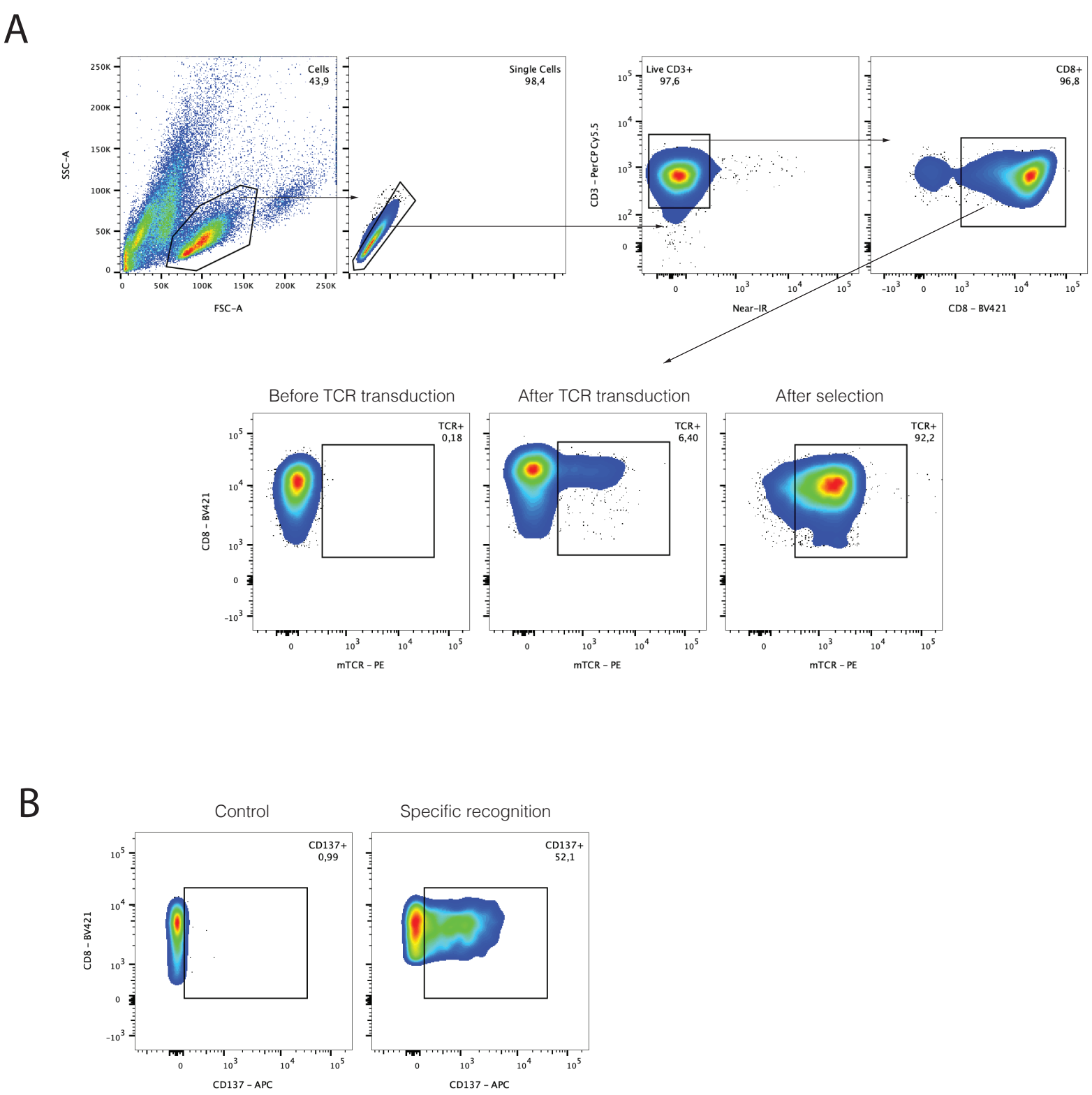

Supplementary figure 4. CD8+ T cells, isolated from healthy donor, engineering to ectopically express TCR via retrotransduction.

A) Representative flow cytometry plots gated on CD8+ T cells isolated from healthy donor PBMC. Expression of the exogenous TCR was analyzed by flow cytometry before and after retroviral transduction, with enrichment of the transduced $CD8^+$ T-cell population confirmed following puromycin selection.

B) Representative flow cytometry plots gated on $CD8^+$ $mTCR^+$ T cells showing specific reactivity following co-culture with target antigen–expressing cells (specific recognition) compared to T cells cultured alone (Control).
