# Supplementary material for: Mismatch Repair–Proficient Colorectal Cancer Can Evade Immune Surveillance through an Intrinsic Suppressive Program
Source: Cancer Discov. 2026 May 13;16(10):1992–2007. doi: 10.1158/2159-8290.CD-26-0542 (PMC13628094; doi:10.1158/2159-8290.CD-26-0542)
Supplement: Supplementary file 9 [file cd-26-0542_supplementary_figure_6_suppsf6.pdf]

Supplementary figure 6.

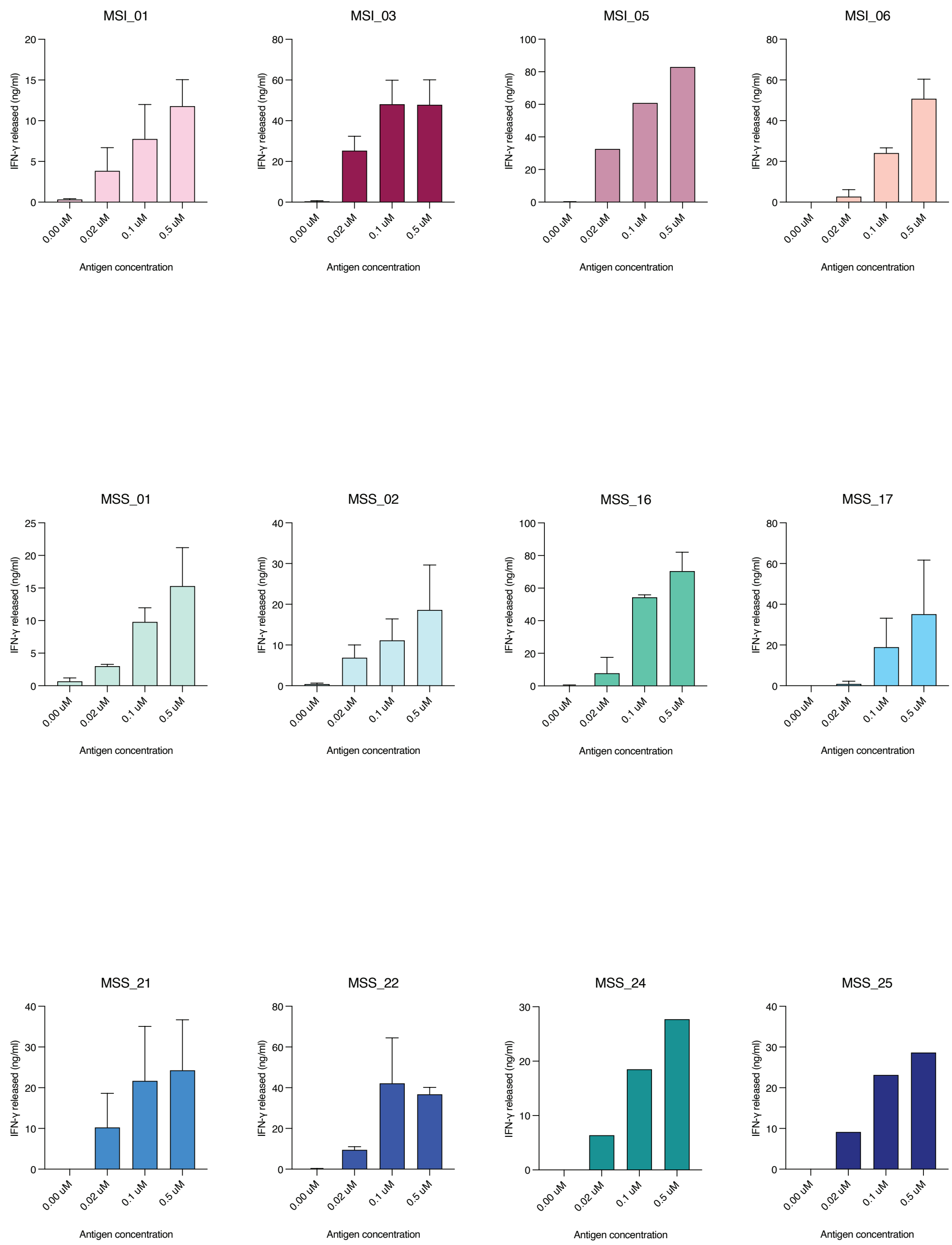

Supplementary figure 6. T cell activation upon coculture with tumor organoids.

Quantification of organoid-induced IFNγ release in the supernatant following exposure to increasing antigen concentrations (0 µM, 0.02 µM, 0.1 µM, and 0.5 µM). Background signal (spontaneous IFNγ expression) was subtracted from each condition. Error bars represent SEM of at least two biological replicates. MSI samples are shown in pink shades, whereas MSS samples are depicted in blue and green shades.
