# Supplementary material for: Mismatch Repair–Proficient Colorectal Cancer Can Evade Immune Surveillance through an Intrinsic Suppressive Program
Source: Cancer Discov. 2026 May 13;16(10):1992–2007. doi: 10.1158/2159-8290.CD-26-0542 (PMC13628094; doi:10.1158/2159-8290.CD-26-0542)
Supplement: Supplementary file 10 [file cd-26-0542_supplementary_figure_7_suppsf7.pdf]

Supplementary figure 7.

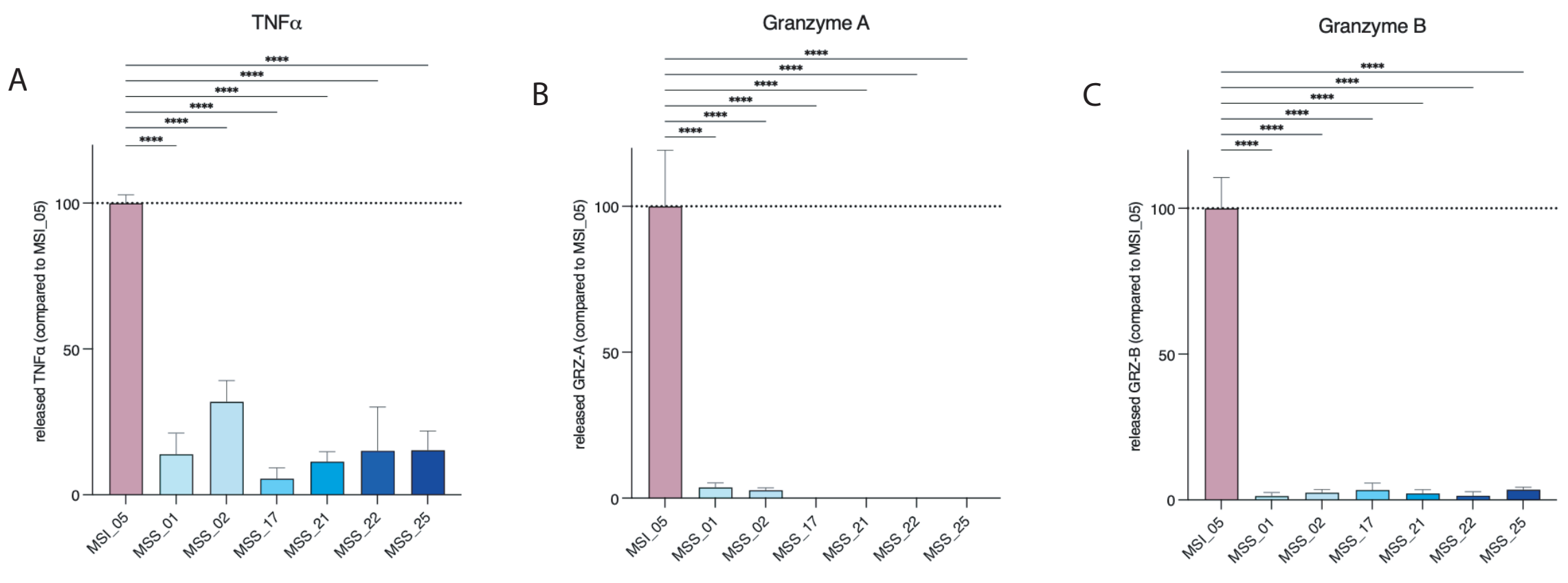

Supplementary figure 7. Comparison between T cell activation triggered by MSI and MSS organoids. Quantification of organoid-induced TNFα (A), Granzyme-A (B) and Granzyme-B (C) release in the supernatant. The background (spontaneous TNFα, Granzyme-A and Granzyme-B production) was subtracted from the signal. Error bars represent SEM of at least two biological replicates. *p<0.05; **p <0.01; ***p<0.001; ****p<0.0001; ns p>0.05 (not shown on the graph); Ordinary one-way ANOVA, multiple comparisons.
