# Supplementary material for: Mismatch Repair–Proficient Colorectal Cancer Can Evade Immune Surveillance through an Intrinsic Suppressive Program
Source: Cancer Discov. 2026 May 13;16(10):1992–2007. doi: 10.1158/2159-8290.CD-26-0542 (PMC13628094; doi:10.1158/2159-8290.CD-26-0542)
Supplement: Supplementary file 11 [file cd-26-0542_supplementary_figure_8_suppsf8.pdf]

Supplementary figure 8.

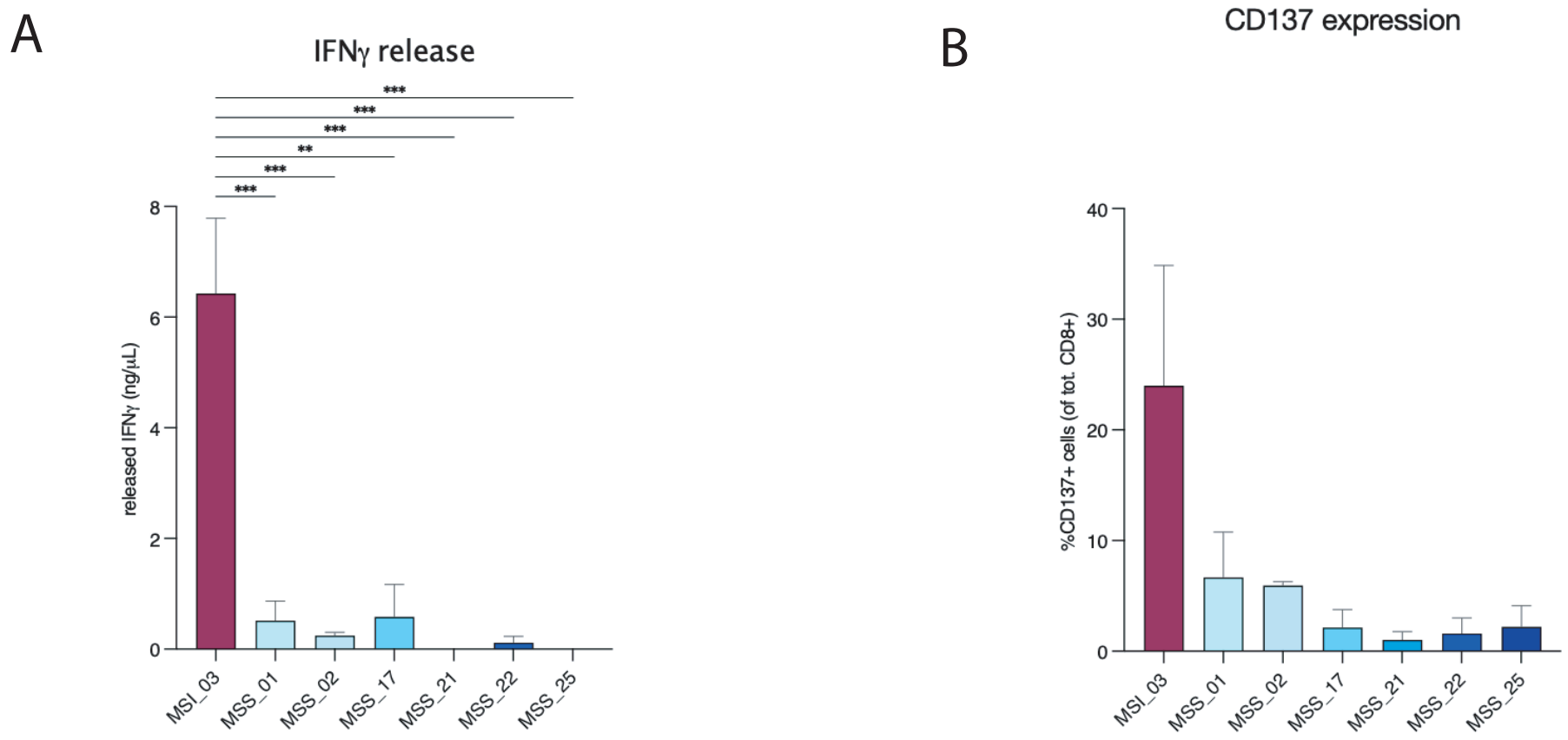

Supplementary figure 8. T cell activation triggered by MSI and MSS organoids using a weakly immunogenic antigen.

Quantification of organoid-induced IFNγ release in the supernatant (A) and CD137 surface expression (B). The background (spontaneous IFNγ release or CD137 expression) was subtracted from the signal. Error bars represent SEM of at least two biological replicates. *p<0.05; **p <0.01; ***p<0.001; ****p<0.0001; ns p>0.05 (not shown on the graph); Ordinary one-way ANOVA, multiple comparisons.
