# Supplementary material for: Mismatch Repair–Proficient Colorectal Cancer Can Evade Immune Surveillance through an Intrinsic Suppressive Program
Source: Cancer Discov. 2026 May 13;16(10):1992–2007. doi: 10.1158/2159-8290.CD-26-0542 (PMC13628094; doi:10.1158/2159-8290.CD-26-0542)
Supplement: Supplementary file 12 [file cd-26-0542_supplementary_figure_9_suppsf9.pdf]

Supplementary figure 9.

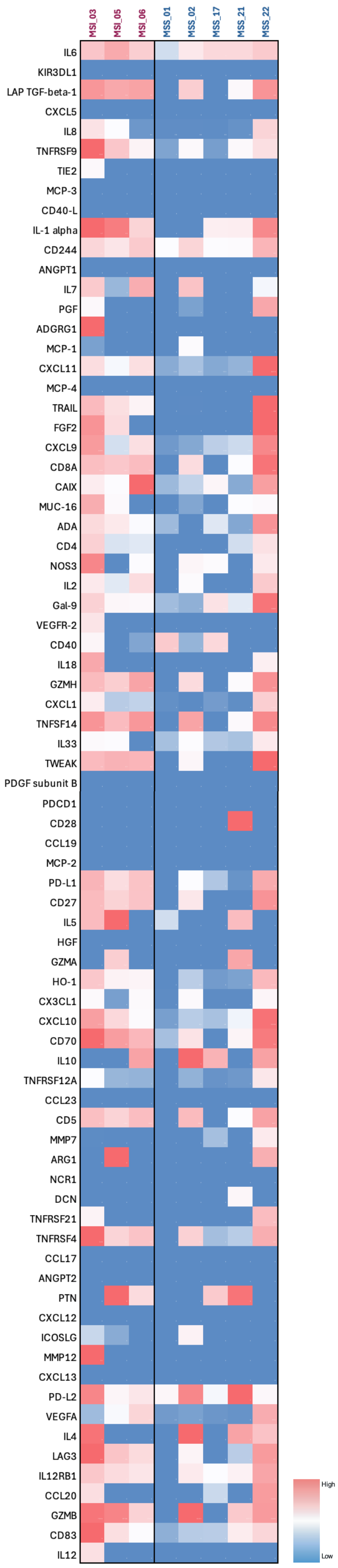

Supplementary figure 9. Secretome profiling

Heatmap showing relative expression levels of a panel of immunomodulatory molecules in the supernatant of MSS- and MSI- PDOs. Colors represent fold changes in immunomodulatory molecule levels (red indicates upregulation, blue indicate downregulation). Columns represent individual patients. Each data point comes from at least 3 technical o biological replicates. Molecules and experimental conditions that have statistically significant abundance between MSS and MSI samples are marked. *$p<0.05$; **$p <0.01$; ns $p>0.05$ (not marked in the figure); Student t-test.
